# Mesenteric and antimesenteric border subregionalization using MR enterography: advancing fibrosis evaluation in Crohn disease

**DOI:** 10.1186/s41747-026-00700-7

**Published:** 2026-03-30

**Authors:** Luyao Wu, Jinjiang Lin, Weikai Zheng, Ruonan Zhang, Qingzhu Zheng, Yangdi Wang, Yaoqi Ke, Ziyin Ye, Xubin Liu, Zhoulei Li, Ren Mao, Zhenpeng Peng, Canhui Sun, Zhihui Chen, Shi-ting Feng, Lili Huang, Xiaodi Shen, Xuehua Li

**Affiliations:** 1 Department of Radiology, The First Affiliated Hospital of Sun Yat-Sen University, Guangzhou, People’s Republic of China; 2 Department of Pathology, The First Affiliated Hospital of Sun Yat-Sen University, Guangzhou, People’s Republic of China; 3 Department of Gastroenterology, The First Affiliated Hospital, Sun Yat-Sen University, Guangzhou, People’s Republic of China; 4 Department of Radiology, The First Affiliated Hospital of Sun Yat-Sen University (Nansha), Guangzhou, People’s Republic of China; 5 Department of Gastrointestinal Surgery, The First Affiliated Hospital, Sun Yat-Sen University, Guangzhou, People’s Republic of China

**Keywords:** Crohn disease, Disease progression, Fibrosis, Magnetic resonance imaging, Printing (three-dimensional)

## Abstract

**Objective:**

Assessment of intestinal fibrosis is an unmet need for patients with Crohn's disease (CD), but spatial heterogeneity of fibrosis may affect accuracy. We developed and validated a novel strategy based on subregionalization into mesenteric and antimesenteric borders on magnetic resonance enterography (MRE).

**Materials and methods:**

We included 184 CD patients across two surgical and one follow-up cohorts. For 12 patients who underwent MRE and surgery (Cohort 1), MRE coregistration with ileal specimens was achieved for 88 sections using three-dimensional-printing and creeping fat information. Optimal multivariable MRE models for mesenteric border, antimesenteric border, and whole-circle regions were constructed referencing histological fibrosis and validated in another 21 patients (Cohort 2). The impact of this strategy on the prediction of disease progression was assessed in a retrospective follow-up cohort of 151 patients (Cohort 3).

**Results:**

Histological fibrosis scores were higher in mesenteric than antimesenteric regions in both surgical cohorts (*p* < 0.001). MRE models showed the highest diagnostic accuracy for fibrosis in the mesenteric border (area under the receiver operating characteristic curve [AUROC] 0.91), followed by the antimesenteric border (AUROC 0.84), and the whole-circle (AUROC 0.77) regions in Surgical Cohort 1. In surgical Cohort 2, MRE also showed higher efficacy in mesenteric (AUROC 0.87) than antimesenteric border (AUROC 0.77). In Cohort 3, baseline fibrosis measurements in the mesenteric border (hazard ratio [HR] 9.25) had the greatest predictive value on disease progression *versus* other regions (HR 0.28‒2.09).

**Conclusion:**

Intestinal fibrosis demonstrates spatial heterogeneity. Subregionalization into mesenteric and antimesenteric borders improves MRE diagnostic power and may aid in predicting CD progression.

**Relevance statement:**

Mesenteric and antimesenteric border subregionalization on MRE advances fibrosis evaluation in CD by addressing the spatial heterogeneity of fibrosis, enabling tailored therapeutic strategies and identifying high-risk patients with disease progression.

**Key Points:**

- Spatial heterogeneity of intestinal fibrosis in CD limits the diagnostic accuracy of conventional whole-region MRE analysis.
- Fibrosis is more severe in the mesenteric than the antimesenteric border, with subregional MRE analysis outperforming whole-circle analysis in fibrosis detection.
- Baseline mesenteric border fibrosis severity most strongly predicts CD progression, necessitating subregionalization for outcome prediction and management.

**Graphical Abstract:**

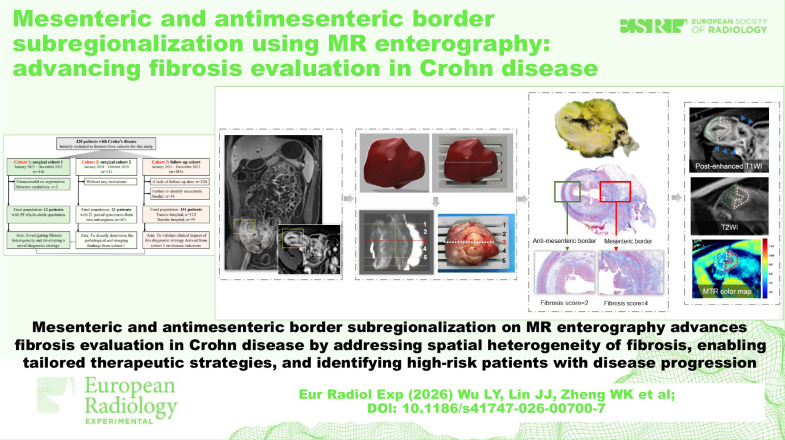

## Background

Intestinal fibrosis, a serious complication of Crohn disease (CD), affects approximately 20‒50% of patients and often leads to fibrostenosis [1], necessitating surgical treatment in the absence of effective antifibrotic therapies [2, 3]. Accurate assessment of fibrosis is essential for optimizing therapeutic strategies.

Magnetic resonance enterography (MRE) has emerged as the preferred non-invasive tool for assessing transmural fibrosis [1, 4]. Several MRI techniques, including magnetization transfer MRI (MTI) [5], diffusion-weighted imaging (DWI) [6], and T1 mapping [7], possess the potential to characterize intestinal fibrosis. However, their diagnostic efficacy varies widely across centers, possibly due to spatial heterogeneity of fibrosis within the gut. This heterogeneity may stem from the chronic progression of CD, where persistent asymmetric transmural inflammation at the mesenteric border rather than the antimesenteric border [8, 9], ultimately culminates in disproportionate fibrosis. Such spatial variation may lead to inconsistent results if not accounted for in diagnostic strategies.

To address this heterogeneity, dividing the bowel into mesenteric and antimesenteric borders based on anatomical differences such as creeping fat [8, 10] can improve diagnostic accuracy. The mesenteric creeping fat index (MCFI) reliably enables this division using cross-sectional imaging [11, 12]. While initially described for CT, the feasibility of deriving MCFI from MRE has been reported [13]. Additionally, layer-by-layer coregistration of MRE with whole-circle specimens, facilitated by three-dimensional (3D)-printing technology [14–16], enhances precision in correlating imaging with histopathology.

Therefore, this study aims to: (1) confirm the presence of spatial heterogeneity in intestinal fibrosis; (2) evaluate the improvement in MRE diagnostic accuracy through subregionalization into mesenteric and antimesenteric borders; and (3) validate the clinical impact of this novel diagnostic strategy on disease outcomes in CD.

## Materials and methods

### Participants

This study, consisting of three cohorts, was approved by our hospital’s institutional research ethics committee (No. [2023]849). Written informed consent was obtained from participants in prospectively enrolled (Cohorts 1 and 2) and waived for those retrospectively included for follow-up evaluation (Cohort 3).

From January 2023 to December 2023, 14 participants were prospectively recruited into Cohort 1 for investigating fibrosis heterogeneity and developing a novel diagnostic strategy using 3D-printing. Inclusion criteria were: (a) CD patients who underwent surgery due to symptomatic terminal ileal strictures (defined as luminal narrowing and wall thickening, with or without upstream dilation [17, 18]); and (b) preoperative MRE within three days before surgery. Exclusion criteria were: (a) failure to identify the mesenteric border; (b) penetrating diseases; or (c) unsuccessful coregistration between modalities. To validate findings from Cohort 1, terminal ileal specimens were prospectively collected from 21 patients (Cohort 2) between January 2024 and October 2024, which adhered to identical inclusion/exclusion criteria for Cohort 1, omitting 3D-printing.

**Fig. 1:**
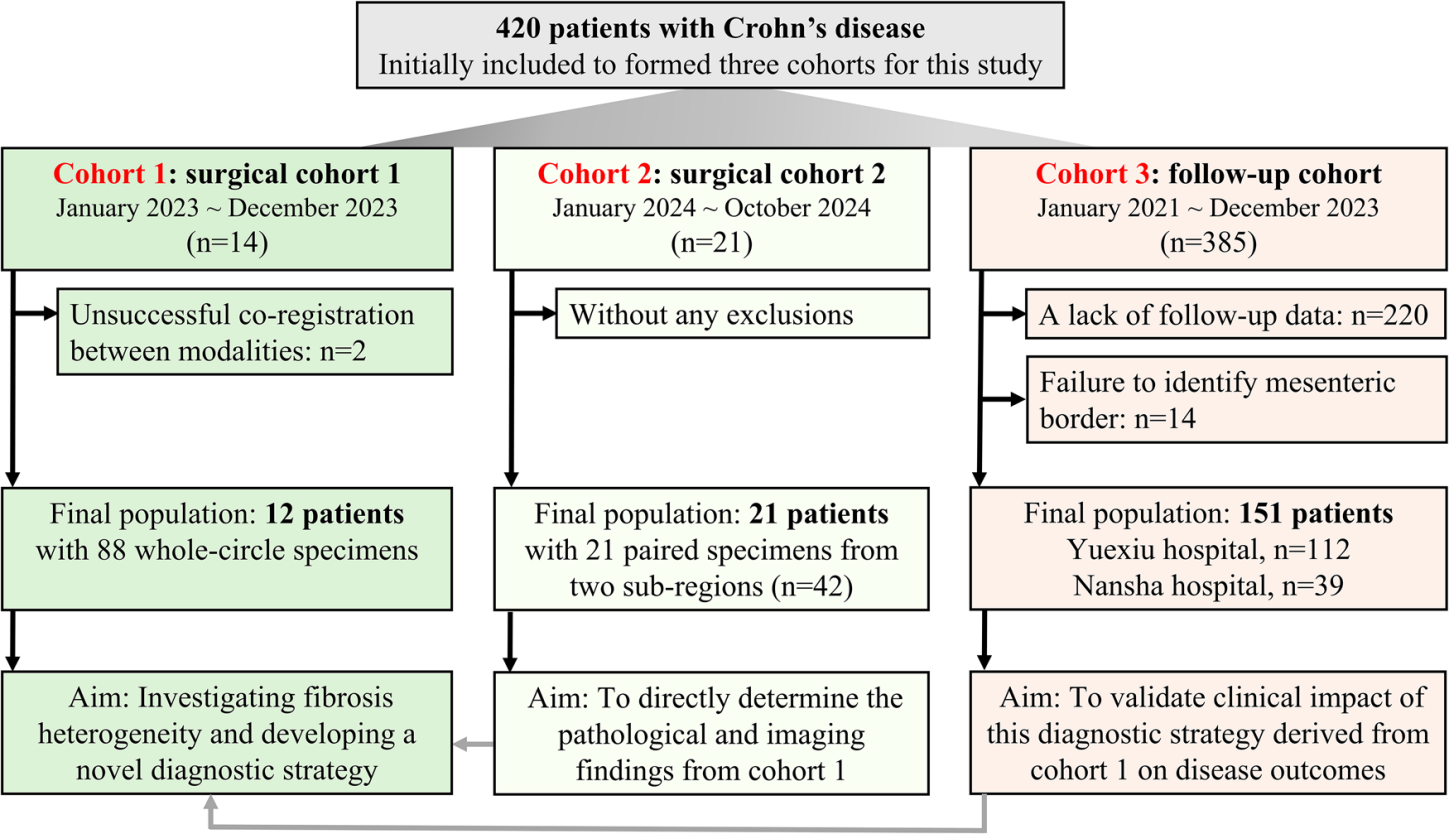
Flow diagram of the study population

Additionally, the clinical impact of this diagnostic strategy on disease outcomes was validated by retrospectively collecting a follow-up cohort of 385 patients from two hospitals (Yuexiu division and Nansha division; January 2021–December 2023) (Cohort 3). Inclusion criteria were: (a) terminal ileal CD without strictures and penetrating diseases at baseline; and (b) a follow-up of at least 12 months for patients unless disease progression occurred. Exclusion criteria were: (a) failure to identify the mesenteric border; or (b) lack of follow-up data (*e.g*., clinical visit records or cross-sectional imaging) for assessing clinical outcome (Fig. 1).

### MRE assessment

All scans were obtained using 3-T scanners (MAGNETOM Prisma; Siemens Healthineers) with standardized sequences: T2-weighted imaging (T2WI), DWI, magnetization transfer imaging (MTI), T1-mapping, unenhanced and contrast-enhanced T1-weighted imaging (T1WI) [5–7] (Supplementary Material 1).

#### Division of mesenteric and antimesenteric borders

**Fig. 2:**
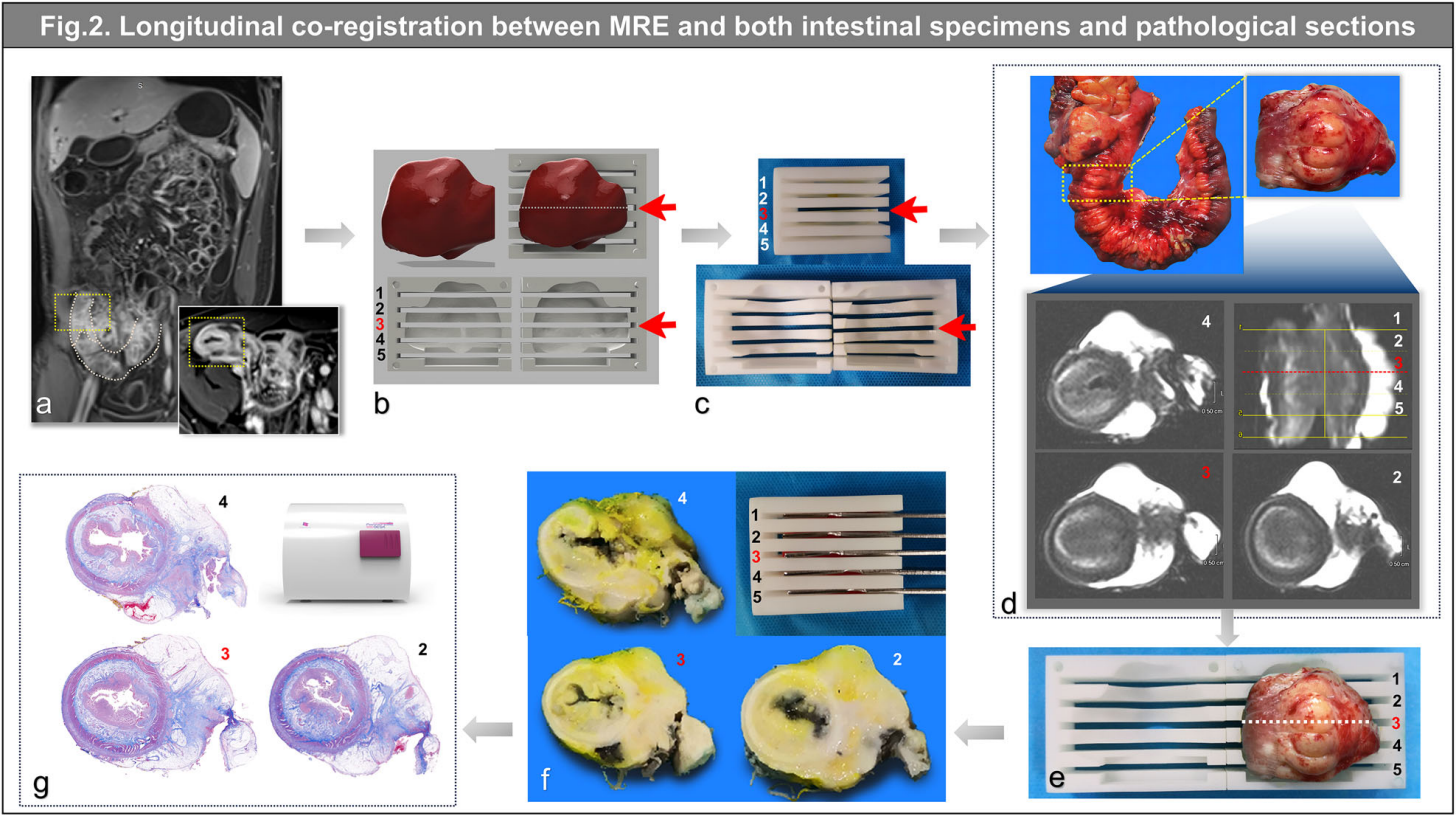
Workflow for coregistration between MRE, specimens, and pathological sections—longitudinal coregistration implemented with 3D-printing. **a** 3D-delineation of the targeted segment (yellow dotted box) on the terminal ileum (pink dotted line) on post-enhanced T1WI (coronal, 3.5 min; axial, 3 min post-contrast administration) in a 32-year-old male patient with CD. **b** Design of a 3D-printing mold with slots based on MRI information to coregister intestinal specimens at the same level. Slots label 1, 3, and 5 correspond to the target intestine’s distal end, narrowest level, and proximal end, respectively. **c** Digital model converted into a tangible object. **d** Surgical obtain and *ex vivo* MRI scan of the specimen. Lines 1–5 on 3D-SPACE-T2WI *ex vivo* correspond to slots 1‒5 on the mold. **e** Repositioning the specimen into a 3D-printing mold using *in vivo* and *ex vivo* information. **f** Sectioning the specimen along the designated slots. **g** Pathological whole-circle-whole-slide imaging of the specimen. Detailed information is shown in Supplementary Material 4. MRE, Magnetic resonance enterography; SPACE, Sampling perfection with application optimized contrast using different flip angle evolution; T2WI, T2-weighted imaging; T1WI, T1-weighted imaging

**Fig. 3:**
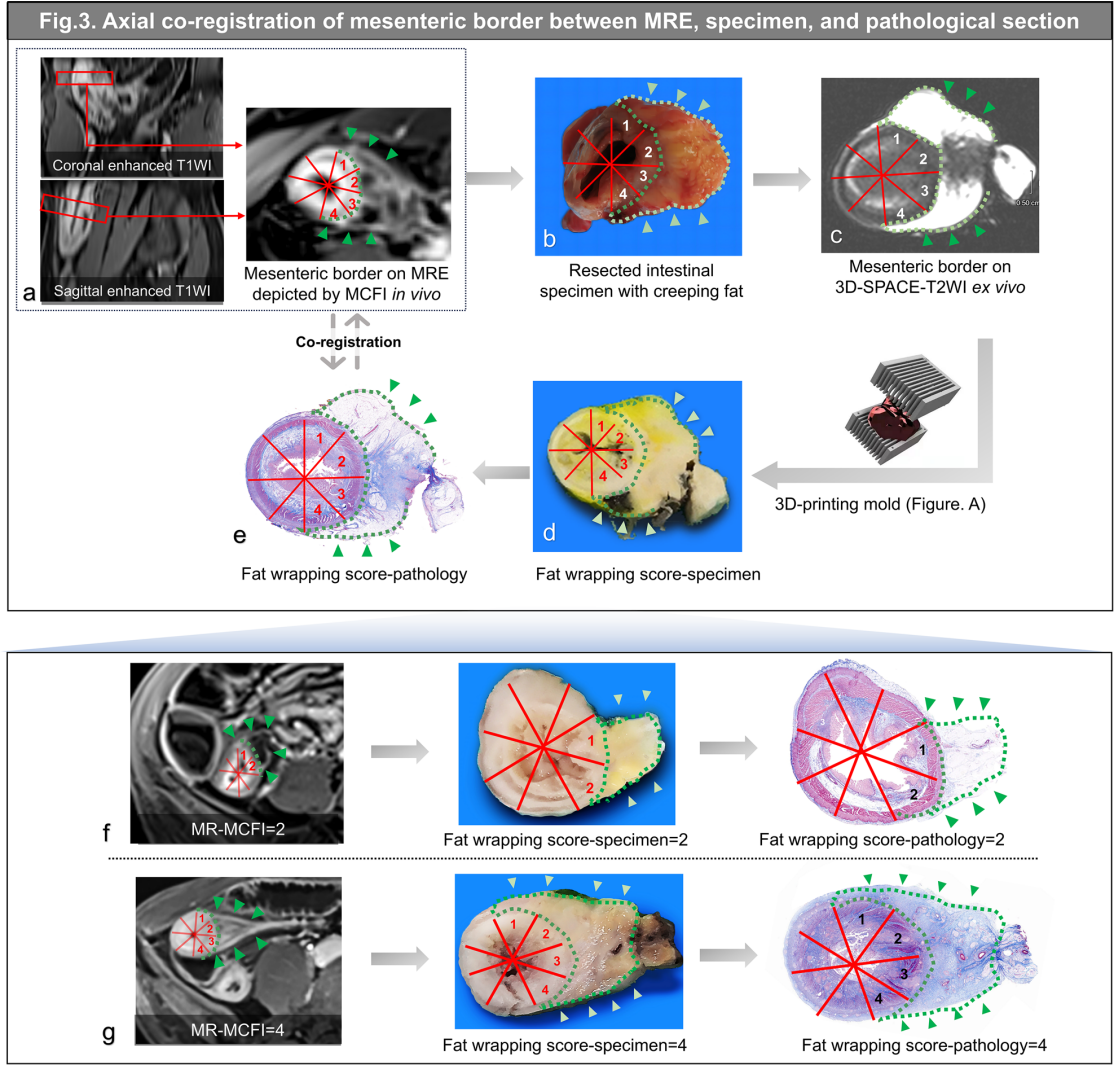
Workflow for coregistration between MRE, specimens, and pathological sections—axial coregistration implemented with creeping fat information. **a** MCFI is reconstructed to identify the mesenteric border (green dotted line) on post-enhanced T1WI at 3 min post-contrast administration (MCFI = 4). **b** Creeping fat (green arrowheads and dotted line) wraps around the resected intestine, resulting in a “fat wrapping score-specimen” of 4. The extent of the mesenteric border in the resected intestine (**b**), as subsequently observed through 3D-SPACE-T2WI *ex vivo* (**c**), along with the specimen (**d**) and pathology (**e**) sections dissected using 3D-printing, demonstrates consistency with MCFI on MRE *in vivo* (**a**). **f**, **g** Another two representative cases demonstrating the accurate coregistration in the mesenteric border among MCFI on MRE, specimens, and pathology using creeping fat information. MRE, Magnetic resonance enterography; SPACE, Sampling perfection with application optimized contrast using different flip angle evolution. T2WI, T2-weighted imaging; MCFI, Mesenteric creeping fat index; T1WI, T1-weighted imaging

Mesenteric and antimesenteric borders were divided based on MCFI, an imaging index scored from 1 to 8 to grade creeping fat wrapping around the gut by assessing the extent of bowel circumference encompassed by vessels in fat *in vivo* [11, 19–23]. Intestinal circumference was divided into eight equal zones, with each vascularized fat-overlapping zone scored as 1. Regions with MCFI ≥ 1 were defined as mesenteric borders; remaining zones constituted antimesenteric borders (Figs. 2 and 3). MCFI was initially reported in CT studies [11, 19–23] and recently applied to MRE [13]. In the current study, we confirmed its feasibility on MRE by comparing it in patients who underwent both MRE and CT (validation results are detailed in Supplementary Material 2). Its postprocessing was conducted on post-enhanced T1WI, and reconstruction quality was scored from 0-to-2 (0 = low; 1 = moderate; 2 = high) by two radiologists (L.W. and Y.W., with two and ten years of experience in abdominal imaging, respectively, and no access to specimens or pathological information; Supplementary Material 2).

#### MRE findings assessment and models development

The axial levels for MRE parameter assessment were selected across all cohorts to ensure precise histology correlation. The process involved identifying the most diseased level of the terminal ileum through a standardized two-plane approach (Fig. 2a, detailed in Supplementary Material 3). Cohort-specific strategies were then applied for parameter evaluation: in Cohort 1, analysis was performed at intervals matching the 3D-printed specimen sections; in Cohort 2, at the single level corresponding to surgical sampling; and in the follow-up cohort (Cohort 3), at the baseline slice with the most pronounced wall thickening. On these selected slices, the six parameters were evaluated across the whole circle, mesenteric border, and antimesenteric border regions as defined by the MCFI (Supplementary Material 3). The subjective assessment items included T2WI signal and DWI signal (0 = isointensity; 1 = mild hyperintensity; and 2 = significant hyperintensity) [24, 25], which were independently performed and collectively assessed by three radiologists to reach consensus (X.L., S.F., and R.Z., with 8‒20 years of experience and no access to specimens or pathological information). Four quantitative parameters, including normalized magnetization transfer ratio (MTR) [5] (normalized MTR = MTR_bowel wall_/MTR_psoas muscle_), apparent diffusion coefficient (ADC), and unenhanced and contrast-enhanced T1 values [7, 26], were measured by X.L. using 2 or 3 regions of interest placed in agreement with other radiologists (Supplementary Material 3); the mean values were used for statistical analysis. Univariable and multivariable models for every region were developed via logistic regression with 5-fold cross-validation and upsampling. The optimal model for each region, chosen for its highest diagnostic accuracy, was implemented as an open calculator application (APP). The structure and usage of this APP are detailed in Supplementary Fig. S5.

### MRE-specimen coregistration

For Cohort 1, three radiologists (X.L., S.F., and R.Z.) collaborated with a senior gastrointestinal surgeon (Z.C.) to identify the extent of the terminal ileum. As per the reported 3D-printing technology [14], contrast-enhanced T1WI were utilized for defining an iso-surface to generate a 3D-printing mold for each intestine by a radiologist (J.L., with 10 years of experience in abdominal imaging). The mold contained slots aligned with axial T2WI images obtained through a half‑Fourier acquisition single‑shot turbo spin-echo sequence, to guide the layer-by-layer coregistration between *in vivo* and *ex vivo* lesions (Figs. 2 and 3). The surgeon carefully resected the whole-circle terminal ileum with adjacent creeping fat, immediately followed by a 3D high-resolution T2WI scan conducted by the radiologist (J.L.) to obtain anatomical information for assisting coregistration between *in vivo* and *ex vivo*. Subsequently, the specimen was restored to its original configuration based on the orientation of MRE and placed within a 3D-printing mold using previously obtained positioning marks. Finally, the whole-circle specimen sections corresponding to MRE were obtained by precise sectioning along the designated slots, to achieve longitudinal coregistration between MRE and specimen (Fig. 2 and Supplementary Material 4). Subsequently, the mesenteric border region of the specimen was delineated using an index, namely, “fat wrapping score-specimen” [11], which was scored from 1 to 8 based on the overlap of creeping fat with each zone in intestinal circumference, divided into eight equal parts by the radiologist (J.L.). The remaining region was defined as the antimesenteric border.

For Cohort 2, we used standard specimen collection procedures, relying on pre-/intra-operative observations for coregistration without 3D-printing. Additionally, small pieces of full-thickness bowel tissue were clipped from both mesenteric and antimesenteric borders at the same axial level rather than obtaining whole-circle specimens (Supplementary Material 4).

### Histopathological assessment

Full-thickness specimens were sectioned (4-µm) and Masson-stained. Fibrotic severity in three regions (mesenteric border, antimesenteric border, and whole-circle) was scored (0-4 [27, 28]) by two blinded pathologists (Z.Y. and X.L., with 5–15 years’ experience), focusing on collagen infiltration depth: 0–2 = none-to-mild; 3–4 = moderate-to-severe [28, 29] (Supplementary Table S1). The mesenteric and antimesenteric border regions on histology were segmented based on creeping fat and quantified by the “fat wrapping score-pathology”, which was consistent with the MCFI-defined borders on MRE to ensure spatial concordance (Fig. 3). The accuracy of this coregistration was evaluated by correlating the MCFI on MRE with the ‘fat wrapping score-pathology’ on histological sections.

Collagen area fraction was quantified using *ImageJ* [30]. Additionally, α-smooth muscle actin (SMA)^+^ immunohistochemistry was performed to quantify fibrogenic cellular activity and complement the fibrosis assessment. The submucosal mesenchymal cells density (area fraction/staining intensity) and muscularis propria thickness were measured on α-SMA-stained sections [31] (Supplementary Figs. S1–S3).

### Impact of subregionalization diagnostic strategy on prediction of clinical outcomes

To validate the clinical value of this subregionalization diagnostic strategy, we compared the impact of baseline fibrosis measurements calculated by the APP from each region on intestinal disease progression in the follow-up cohort (Cohort 3), as fibrosis potentially results in adverse outcomes [32]. Intestinal disease progression was defined as the development of penetrating disease, stricture/obstruction (luminal narrowing, wall thickening, and upstream dilation), or CD-related surgery [33]. The follow-up endpoint was defined as the time of disease progression or the end of follow-up for patients without disease progression. The disease-progression-free survival time was defined as the time from baseline to follow-up endpoint.

### Statistical analysis

#### Sample size

Calculations were performed with PASS 2023 (NCSS, LLC). For Surgical Cohort 1, 64 bowel segments (32 per fibrosis severity group) were required to achieve 80% power (one-sided α = 0.05) to detect an area under receiver operating characteristic curve (AUROC) improvement from 0.700 (null) [28] to 0.850 (alternative), assuming a standard deviation (SD) ratio of 0.8 and correlation of 0.6 [34]. Surgical Cohort 1 exceeded this requirement (88 segments: 54 non-mild, 34 moderate-severe fibrosis). Surgical Cohort 2 was designed as an independent validation set to test the generalizability of Surgical Cohort 1’s findings; its sample size was determined by the availability of specimens meeting identical inclusion criteria during the study period. For the Follow-up Cohort, 130 patients were needed to achieve 80% power (one-sided α = 0.05) to detect a hazard ratio of 2.0 for disease progression (assumed event rate = 40% [35], covariate SD = 0.5) [36]. Our enrollment of 151 patients met this threshold.

### Diagnostic performance

Statistical analysis used Python 3.12.0. Statistical significance was defined as a two-sided *p*-value < 0.05, with *p* < 0.10 indicating marginal significance. Quantitative data were expressed as mean ± standard deviation, and qualitative data were presented as medians (interquartile ranges [IQRs]). One-way ANOVA or Mann–Whitney *U*-test was used to detect differences in data among different groups. Interobserver agreement for the subjective T2WI and DWI signal intensity scores was assessed using the intraclass correlation coefficient for absolute agreement based on a two-way random-effects model, calculated separately for each cohort and overall. Spearman correlation analysis was used to analyze their correlations. Model performance was evaluated via AUROC and visualized by SHapley Additive exPlanations [SHAP] [37], and Youden-index thresholds. AUROCs were compared using the DeLong test. Univariate and multivariate Cox regression analysis were used to identify independent risk factors for disease progression. The Kaplan–Meier method with log-rank tests was used to assess differences in disease-progression-free survival.

## Results

### Baseline characteristics

Cohort 1 finally included 12 participants, from whom we obtained 88 pathological sections according to MRE and 3D-printing. The number of sections per patient ranged from five to eleven, depending on the length of the target ileum (37.19 ± 8.49 mm). All 21 participants recruited for Cohort 2 were included without any exclusions. They provided 21 paired specimens (*n* = 42) from mesenteric and antimesenteric border regions. In the follow-up cohort, Cohort 3 (initially *n* = 385), 234 were excluded (lack of follow-up: *n* = 220; undetermined mesenteric border: *n* = 14), resulting in a final inclusion of 151 patients (Yuexiu division, *n* = 112; Nansha division, *n* = 39) (Fig. 1). Clinical characteristics are shown in Table 1.

**Table 1:** Demographic and clinical characteristics of the participants

Characteristics	Cohort 1 (*n* = 12)	Cohort 2 (*n* = 21)	Cohort 3 (*n* = 151)
Sex (males/females)	10/2	18/3	110/41
Age (years)	32.42 ± 7.82	30.95 ± 11.56	30.47 ± 9.59
Body mass index (kg/m^2^, mean ± SD)	17.72 ± 2.49	19.19 ± 4.12	20.14 ± 3.04
Smoking history, *n* (%)	2/12 (16.67%)	2/21 (9.52%)	15/151 (9.93%)
Disease duration (months, mean ± SD)	61.50 ± 54.52	70.37 ± 30.61	58.25 ± 43.18
Disease location			
L1 (ileal)	0	0	24
L2 (colonic)	0	0	0
L3 (ileocolonic)	12	21	127
CD activity index, *n* (%)			
Remission (< 150)	2/12 (16.67%)	5/21 (23.81%)	53/151 (35.10%)
Mild disease (150‒220)	4/12 (33.33%)	6/21 (28.57%)	44/151 (29.14%)
Moderate disease (220‒450)	5/12 (41.67%)	9/21 (42.86%)	52/151 (34.44%)
Severe disease (> 450)	1/12 (8.33%)	1/21 (4.76%)	2/151 (1.32%)
C-reactive protein (mg/L, median [IQR])	9.75 [5.40, 29.43]	9.39 [3.96, 27.60]	5.04 [1.20, 23.15]
Erythrocyte sedimentation rate (mm/h, mean ± SD)	40.67 ± 24.53	39.86 ± 25.72	28.33 ± 22.43
Medicine use before/at baseline^#^, *n* (%)			
Biologics	10/12 (83.33%)	16/21 (76.19%)	128/151 (84.77%)
Corticosteroids	4/12 (33.33%)	6/21 (28.57%)	32/151 (21.19%)
Immunomodulator	8/12 (66.67%)	9/21 (42.86%)	58/151 (38.41%)
Intestinal disease progression^*^, *n* (%)			
Strictures	-	-	50/55 (90.91%)
Penetrating diseases	-	-	25/55 (45.45%)
Surgery	-	-	47/55 (85.45%)

Data are presented as mean ± SD for data that follow a normal distribution, or as median (IQR) for data that do not follow a normal distribution

*SD* Standard deviation, *IQR* Interquartile range

^#^ The same patient may receive multiple medications prior to or at baseline, resulting in the cumulative number of these three medications exceeding the total number of patients in each cohort

^*^ The same patient may experience multiple intestinal disease progressions at the end of follow-up; hence, the combined number of the three adverse outcomes shown here exceeds the total number of patients with intestinal disease progression

### Interobserver agreement for subjective MRE assessments

The inter-observer agreement for the subjective assessment of T2WI and DWI signal intensities was excellent across all cohorts. As detailed in Supplementary Material 3, the intraclass correlation coefficient values for T2WI signal assessment were 0.93 in Cohort 1, 0.79 in Cohort 2, 0.83 in Cohort 3, and 0.89 for the overall population. Similarly, for DWI signal assessment, intraclass correlation coefficient values were 0.94, 0.79, 0.83, and 0.86, respectively.

### MCFI reconstruction on MRE is feasible

MRE-based MCFI were significantly correlated with CTE-based MCFI (*r* = 0.96, 0.89, both *p* < 0.001; Supplementary Material 2). Using creeping fat on gross specimens as a reference standard in Cohort 1, MRE-based MCFI accurately depicted the extent of creeping fat, showing a significant correlation with fat wrapping score-specimen (*r* = 0.88, *p* < 0.001; Figs. 2 and 3). MCFI reconstruction quality was consistently rated high across two surgical cohorts (both median scores = 2, IQR [2,2]) and Cohort 3 (median score = 2, IQR [1, 2]).

### Mesenteric borders were coregistered accurately across three modalities using 3D-printing and MCFI

To assess the accuracy of longitudinal and axial coregistrations across modalities, we performed correlation analysis on their scores in Cohort 1. MCFI, fat wrapping score-specimen/-pathology all exhibited consistent median [IQR] values of 4 [3, 4], ranging from 1 to 7. The scores between specimens and pathology exhibited complete consistency. MCFI correlated robustly with fat wrapping pathology scores (*r* = 0.88, *p* < 0.001).

### Histological fibrosis varies across different regions

**Fig. 4:**
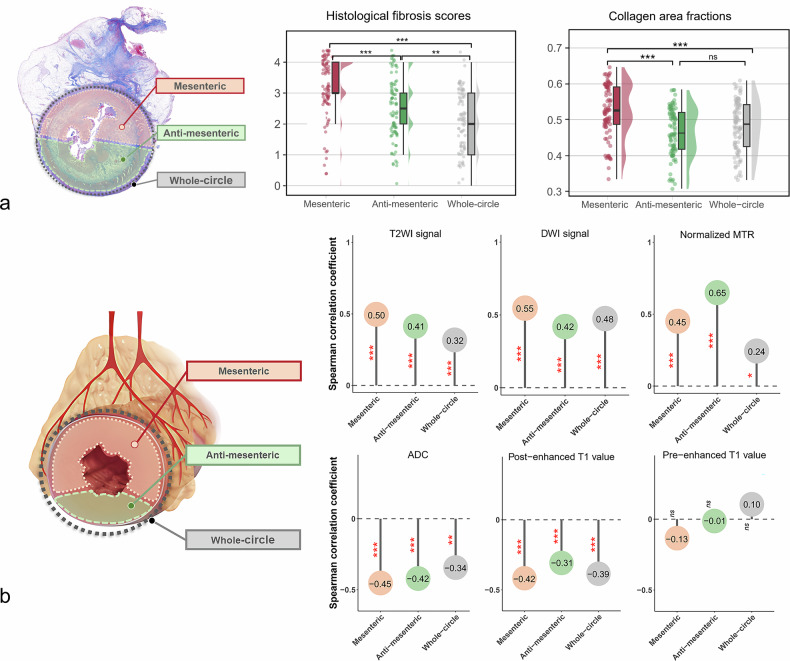
Histological fibrosis varies among the mesenteric border, antimesenteric border, and whole-circle regions, and the correlation between MRE parameters and histological fibrosis improves after subregionalization in surgical Cohort 1. **a** The histological fibrosis scores and collagen area fractions in the mesenteric border region are higher than those in the antimesenteric border and whole-circle regions, indicating the presence of spatial heterogeneity of fibrosis. The raincloud plot combines an illustration of the data distribution (the ‘cloud’) with jittered raw data (the ‘rain’) and a boxplot. The boxplot represents the IQRs (25th through 75th percentiles, box), median values (50th percentile, bar within the box), and 5th and 95th percentiles (whiskers below and above the box). ^**^p < 0.01; ^***^p < 0.001; ns, not significant. **b** After subregionalization, correlations of MRE parameters with histological fibrosis scores are improved in both mesenteric and antimesenteric border regions, compared to the whole-circle region. The length of the line on the lollipop plot represents correlation strength, with a longer line indicating a stronger correlation. ^*^p <  0.05; ^**^p <  0.01; ^***^p <  0.001. ns, Not significant; MRE, Magnetic resonance enterography; T2WI, T2-weighted imaging; DWI, Diffusion-weighted imaging; MTR, Magnetization transfer ratio

To determine fibrosis heterogeneity, histological fibrosis-related alterations were compared across three regions in Cohort 1. Longitudinally, histological fibrosis scores in the same segment varied at multiple slices (Supplementary Table S2). Axially, fibrosis scores were highest in mesenteric border (median [IQR], 3 [3, 4]), followed by antimesenteric border (2.5 [2, 3]) and whole-circle regions (2 [1, 3]) (*p* = 0.000‒0.004; Fig. 4a). Similarly, collagen area fraction in mesenteric border (0.52 ± 0.07) was higher than antimesenteric border (0.47 ± 0.07) and whole circle regions (0.48 ± 0.07) (both *p* < 0.001; Fig. 4a). No significant difference in this fraction was found between the latter two (*p* = 0.382). Submucosal α-SMA^+^ area fractions, submucosal α-SMA^+^ staining intensity, and the mean thickness of muscularis propria exhibited a trend of being highest in mesenteric border, followed by whole-circle and antimesenteric border regions; however, these differences did not reach statistical significance (*p* = 0.243‒0.716; Supplementary Table S3).

In Cohort 2, fibrosis scores in the mesenteric border were also higher (4 [3, 4]) than the antimesenteric border (3 [2, 3]) (*p* = 0.013), re-confirming the presence of fibrosis heterogeneity.

### Correlation between MRE and histological fibrosis are improved after subregionalization

Histological fibrosis heterogeneity prompted us to investigate whether MRE could enhance its diagnostic efficacy via subregionalization measurement. Here, fibrosis scores were used as a reference standard for fibrosis severity.

In Cohort 1, except for pre-enhanced T1 values, the other five parameters significantly correlated with fibrosis scores (Fig. 4b). After subregionalization, correlation of these five parameters improved in both mesenteric (|*r*| = 0.42–0.55, all *p* < 0.001) and antimesenteric (|*r*| = 0.31‒0.65, all *p* < 0.001) border regions, compared to whole-circle region (|*r*| = 0.24‒0.48, *p* = 0.000‒0.022). Among them, four parameters (*i.e*., T2WI signal, DWI signal, ADC, and post-enhanced T1 values) had stronger correlations with fibrosis in the mesenteric border region than other regions, which was further confirmed by the results from Cohort 2 (Supplementary Table S4).

### MRE offers the greatest diagnostic efficacy for fibrosis in the mesenteric border among the three regions

To address the diagnostic challenge from fibrosis heterogeneity, we developed univariable and multivariable MRE models for assessing fibrosis in different regions and compared their abilities, with the fibrosis score as the reference standard.

**Fig. 5:**
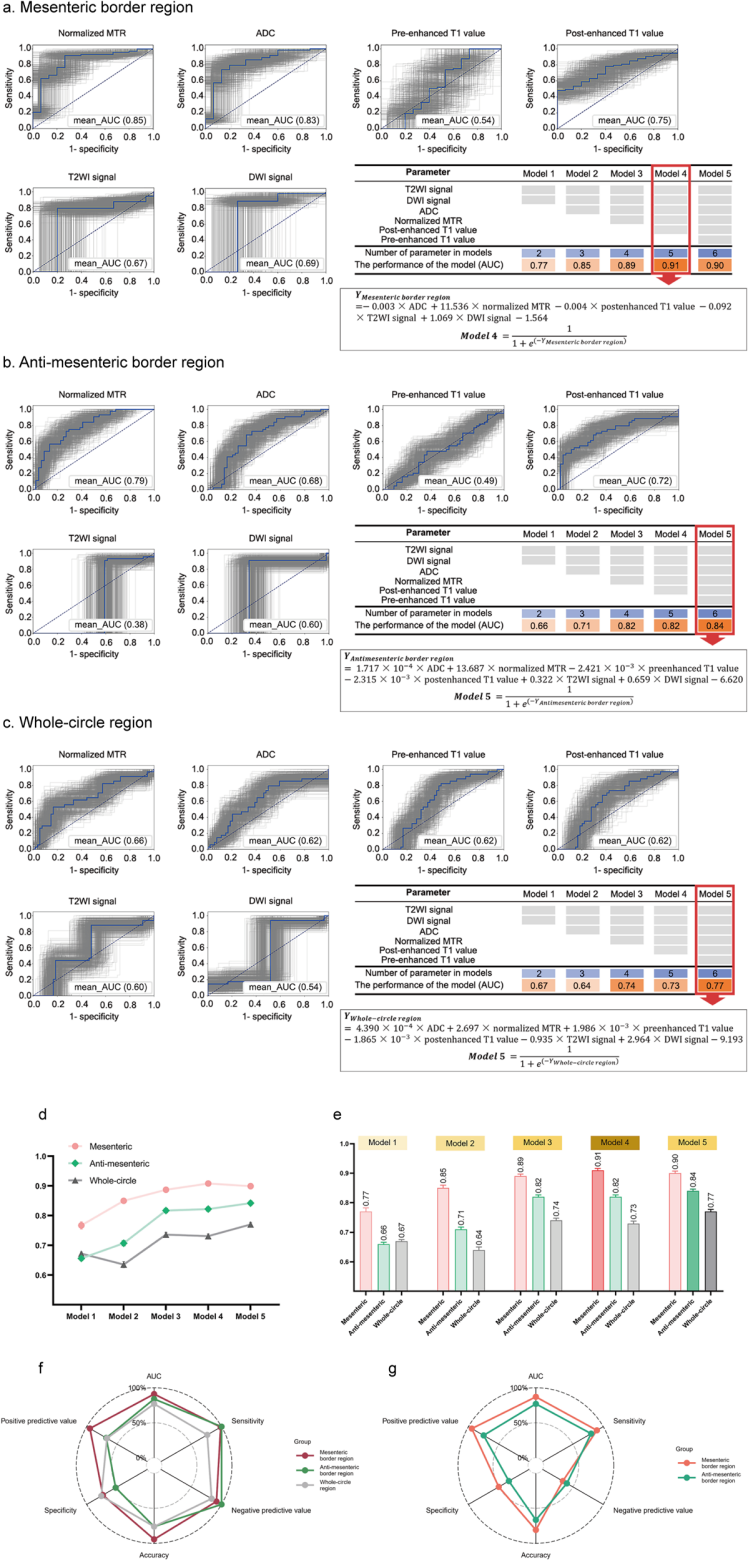
Diagnostic performance of MRE in distinguishing non-mild from moderate-severe intestinal fibrosis in mesenteric border, antimesenteric border, and whole-circle regions in both surgical cohorts (Cohorts 1 and 2). Diagnostic performance of univariable and multivariable MRE models in mesenteric (**a**), antimesenteric (**b**), and whole-circle (**c**) regions in Surgical Cohort 1 is shown. The optimal multivariate model for assessing fibrosis in each region is also presented. Their cut-off values were 0.51, 0.27, and 0.44, respectively. In surgical cohort 1, the AUROC of each type of multivariate model is highest in the mesenteric border region, followed by the antimesenteric border and whole-circle regions (**d**, **e**). Most other performance evaluation parameters, such as accuracy, sensitivity, specificity, positive predictive value, and negative predictive value, demonstrate a similar trend on a radar map (**f**). In surgical cohort 2, optimal MRE models in the two subregions still show satisfactory diagnostic performance. Among them, the AUROC, accuracy, sensitivity, specificity, and positive predictive value of the optimal MRE model in the mesenteric border region are higher than those in the antimesenteric border region (**g**). MRE, Magnetic resonance enterography; AUROC, Area under the receiver operating characteristic curve; T2WI, T2-weighted imaging; DWI, Diffusion-weighted imaging; MTR, Magnetization transfer ratio; ADC, Apparent diffusion coefficient

In Cohort 1, normalized MTR showed the greatest diagnostic efficacy in distinguishing none-to-mild from moderate-to-severe fibrosis in the mesenteric border region (AUROC = 0.85), compared to other univariable models (AUROCs = 0.54‒0.83; Fig. 5a and Supplementary Table S5). These six parameters were incrementally integrated to construct multivariable models (Models 1–5). Among them, Model 4 showed the highest AUROC of 0.91, surpassing other multivariable models (AUROCs = 0.77‒0.90) (Fig. 5a and Supplementary Table S6). In antimesenteric border (Fig. 5b and Supplementary Table S7) and whole-circle (Fig. 5c and Supplementary Table S8) regions, multivariable models ranging from Models 1–5 were also observed to continuously improve diagnostic ability.

Overall, the diagnostic efficacy of each type of multivariable model was highest in the mesenteric border, followed by the antimesenteric border and whole-circle regions (Fig. 5d, e and Supplementary Table S9). The optimal model in the mesenteric border region was Model 4 (AUROC = 0.91). In antimesenteric border (AUROC = 0.84) and whole-circle regions (AUROC = 0.77), Model 5 was the optimal choice (Fig. 5f, Supplementary Material 5, and Supplementary Fig. S4). These optimal models were utilized in Cohort 2 and consistently demonstrated satisfactory diagnostic efficacy, exhibiting superior performance in the mesenteric border (AUROC = 0.87) than the antimesenteric border (AUROC = 0.77; Fig. 5g).

**Fig. 6:**
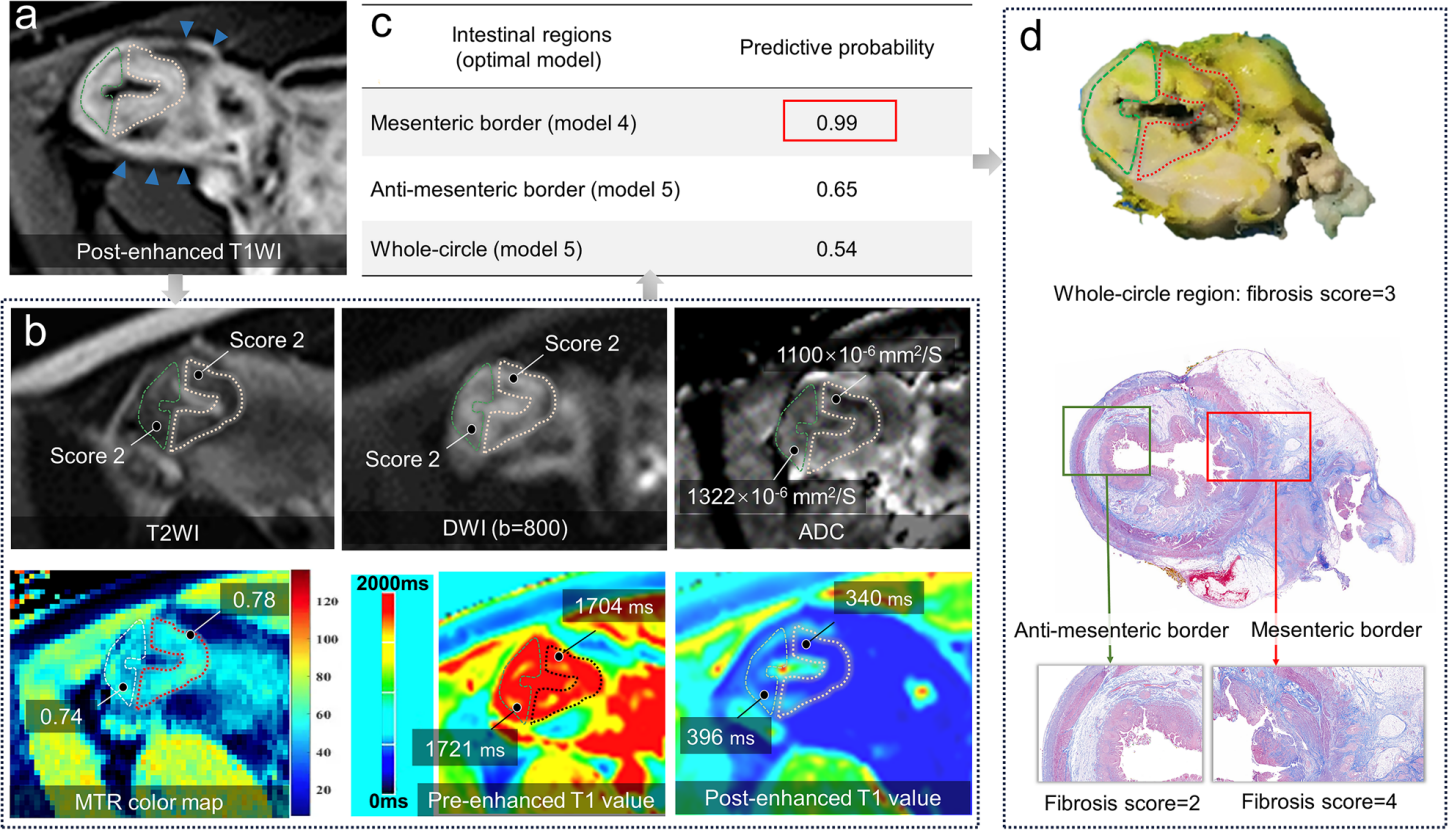
A representative case demonstrating the improved performance of MRE using a subregionalization diagnostic strategy. In the same patient as shown in Fig. 1, the division of mesenteric border (pink dotted line) and antimesenteric border (green dotted line) regions are performed according to MCFI (blue arrowheads) on axial post-enhanced T1WI (**a**) and are subsequently copied to other maps (**b**). The six MRE parameters in the whole-circle region are as follows: T2WI = 2, normalized MTR = 0.76, DWI = 2, ADC = 1311 × 10^-6^ mm^2^/S, pre-enhanced T1 value = 1,713 ms, post-enhanced T1 value = 395 ms. While their measurements in subregions are presented in the figures (**b**). The optimal multivariate MRE models corresponding to each region on APP are used for assessing fibrosis (**c**). Using pathological findings as a reference standard (**d**), the diagnostic accuracy of MRE improves significantly after subregionalization, particularly in the mesenteric border region (**c**). This diagnostic process, using an optimal MRE model in the mesenteric border region on APP, is also presented in Supplementary Fig. S5. MRE, Magnetic resonance enterography; MCFI, Mesenteric creeping fat index; T1WI, T1-weighted imaging; T2WI, T2-weighted imaging; DWI, Diffusion-weighted imaging; MTR, Magnetization transfer ratio; ADC, Apparent diffusion coefficient

The optimal models were integrated into an APP calculator (available at: https://github.com/SchwarzW/APP-based-calculator-for-assessing-fibrosis-in-different-intestinal-subregions; Supplementary Fig. S5), and its effectiveness to address the diagnostic challenge posed by fibrosis heterogeneity is shown in a representative case (Fig. 6).

### Prediction of the clinical outcome of baseline fibrosis measurement in the mesenteric border region is greater than that in other regions

**Fig. 7:**
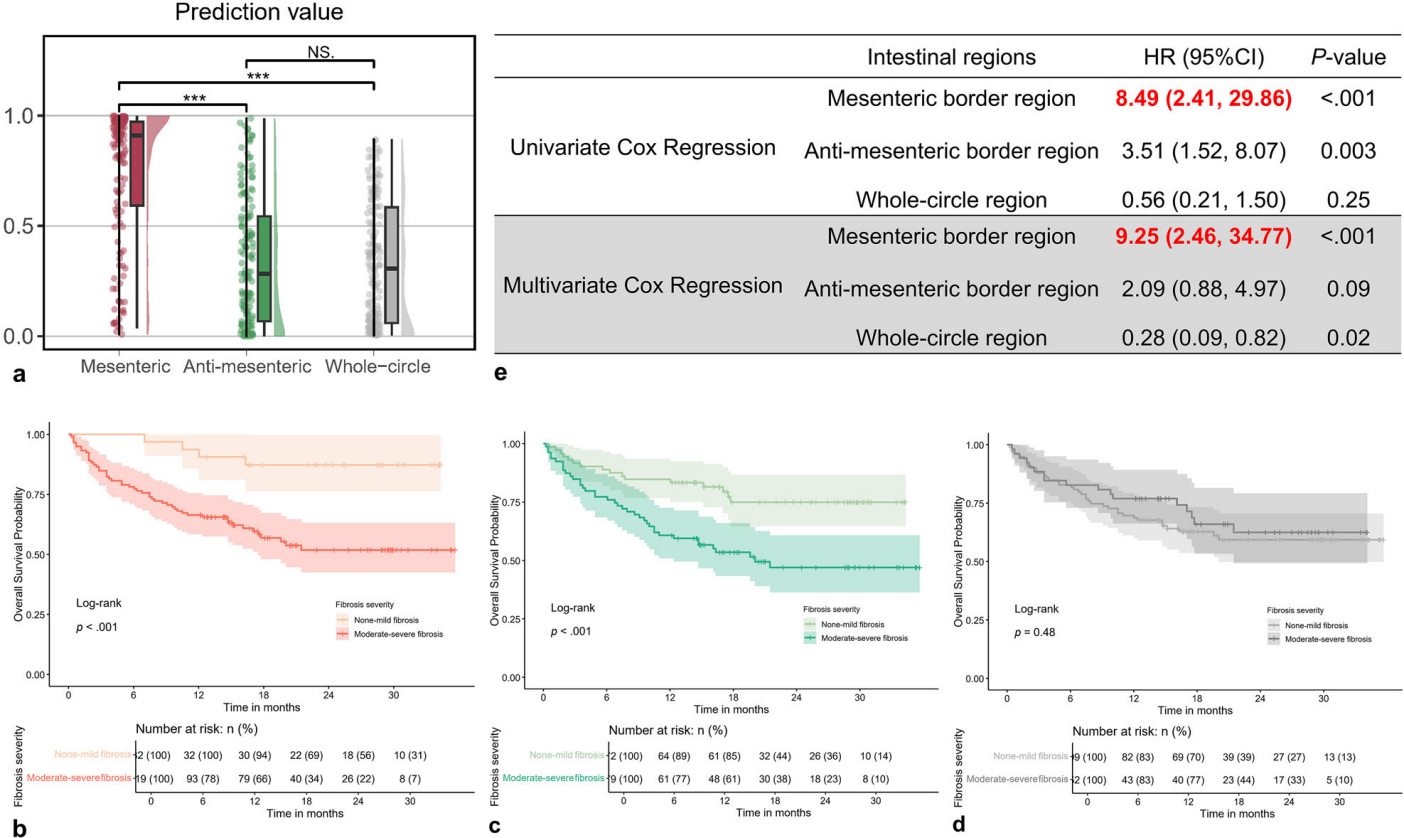
The impact of baseline fibrosis measurement in the mesenteric border region on clinical outcome is greater than in other regions. In the follow-up cohort, the baseline fibrosis measurement in the mesenteric border region is significantly higher than that in both the antimesenteric border and the whole-circle regions (**a**). After subregionalization, using the mesenteric border measurement for Kaplan–Meier analysis, the median disease-progression-free survival of patients with moderate-to-severe fibrosis was significantly shorter than that of patients with none-to-mild fibrosis (**b**). The use of antimesenteric border measurement (**c**) yields a similar but relatively attenuated result, whereas the employment of whole-circle value (**d**) does not produce a comparable result. In both univariate and multivariate Cox regression analysis, baseline mesenteric border measurement is the most important independent risk factor of disease progression among all regions (**e**). The interpretation of the raincloud plot can be found in Fig. 4. ^***^p < 0.001. ns, Not significant; HR, Hazard ratio

To validate the clinical value of this subregionalization diagnostic strategy, we retrospectively followed up another cohort of CD patients. Overall, 55 out of 151 patients experienced disease progression during a median follow-up period of 16.34 [9.92, 25.83] months. Using APP calculator for fibrosis degrees, the predictive value in mesenteric border was higher than both antimesenteric border and whole-circle regions (both *p* < 0.001; Fig. 7a). According to Kaplan–Meier analysis, using baseline mesenteric border values, the median disease-progression-free survival of patients with moderate-to-severe fibrosis was significantly shorter than those with none-to-mild fibrosis (*p* < 0.001; Fig. 7b). The same result was observed when using antimesenteric border values (*p* < 0.001; Fig. 7c), but not whole-circle values (*p* = 0.48; Fig. 7d). In both univariate and multivariate Cox regression analysis, baseline mesenteric border measurement was identified as the most important independent risk factor of disease progression among all regions (hazard ratio [HR] (95% confidence interval) = 8.49 (2.41, 29.86), 9.25 (2.46, 34.77), respectively; all *p* < 0.001; Fig. 7e).

## Discussion

In this study, in CD patients, fibrotic severity was greater in the mesenteric border than in the antimesenteric border region, confirming spatial heterogeneity of fibrosis and emphasizing the necessity for subregional evaluation. After subregionalization, the diagnostic efficacy of MRE for fibrosis improved in both regions, particularly the mesenteric border region. Optimal multivariable MRE models were developed and validated to address heterogeneity-driven diagnostic challenges. Baseline fibrosis measurements in the mesenteric border region had the greatest impact on clinical outcomes for CD compared to other regions, supporting the clinical value of this subregionalization diagnostic strategy.

Prior studies primarily focused on longitudinal-axis fibrosis progression linked to creeping fat (*e.g.*, extent of fat proliferation or tissue stiffness) [10, 11, 38], while transverse-axis heterogeneity remains poorly characterized. Our results identified asymmetric collagen deposition depth, α-SMA^+^ mesenchymal cell infiltration, and muscularis propria hypertrophy between mesenteric and antimesenteric borders at identical axial levels. Conventional whole-wall assessment methods (*e.g.*, random region-of-interest mapping) may underestimate fibrosis severity due to this spatial variability, potentially delaying timely interventions and exacerbating complications [3]. Therefore, it may be more appropriate to evaluate fibrosis separately in mesenteric and antimesenteric border regions. Particularly, mesenteric border region assessment can better reflect the maximal fibrosis severity, crucial for guiding treatment decisions.

MRE is an optimal imaging tool for evaluating transmural fibrosis, providing superior soft-tissue contrast and avoiding ionizing radiation compared to CT [1]. After subregionalization, the diagnostic accuracy of MRE parameters for fibrosis within both mesenteric and antimesenteric border regions was enhanced. These results supported our hypothesis that the impact of fibrosis heterogeneity on the diagnostic accuracy of MRE can be minimized by implementing a subregionalization strategy. Univariable MRE parameters showed higher accuracy in the mesenteric border, while multivariable models further refined diagnostic precision and were validated in an independent surgical cohort, thereby effectively addressing the diagnostic challenge arising from this heterogeneity.

This subregionalization diagnostic strategy is readily integrable into routine clinical practices, as radiologists can reliably identify the mesenteric border on MRE by tracing mesenteric vessels toward the bowel wall. For CD patients with multiple strictures, fibrosis measurements in the mesenteric border region using APP can help develop individualized treatment strategies for each stricture: surgical resection for strictures with predominant moderate-to-severe fibrosis *versus* anti-inflammatory therapy for those with none-to-mild fibrosis [2, 3]. Additionally, baseline mesenteric border region fibrosis was the strongest predictor of disease progression among all regions, suggesting that the subregionalization diagnostic strategy offers more precise stratified information for CD patients. More severe fibrosis in this specific subregion may lead to adverse outcomes for the gut, as its heavier fibrosis burden increases local rigidity, creating luminal pressure gradients that predispose to stricture/fistula development. This finding underscores the clinical imperative of the subregionalization diagnostic strategy.

Our study had certain advantages. Accurate layer-by-layer coregistration between MRE and the specimen has been a challenging task. We used 3D-printing to ensure the optimal alignment. MCFI on MRE showed high consistency with fat wrapping score-pathology, aligning with a prior study [11]. This result ensured accurate layer-by-layer and subregional coregistration between modalities, aided by 3D-printing, improving the reliability of findings derived from Cohort 1. Additionally, we analyzed not only collagen changes but also the alterations in α-SMA^+^ mesenchymal cells and muscularis propria hypertrophy, providing more pathological evidence for assessing fibrosis heterogeneity. Another key strength of our study was the validation of our diagnostic strategy across the disease severity spectrum. Its ability to predict outcomes in early-stage patients (Cohort 3), despite being developed in an advanced-disease cohort (Cohorts 1 and 2), powerfully enhanced its potential clinical translatability and generalizability.

This study has limitations. First, despite demonstrating improved diagnostic accuracy and clinical utility, the relatively small sample size, particularly in the prospective surgical cohorts, might limit generalizability. Second, the follow-up cohort utilized for assessing clinical utility had a retrospective nature, introducing potential biases inherent to such study designs. Third, we did not systematically quantify the degree of inflammation in all histological specimens; although our multi-parametric approach and validation across cohorts support the robustness of our findings, the potential influence of coexisting inflammation on some MRE parameters cannot be entirely ruled out. Fourth, while the use of a standard specimen obtain technique in Cohort 2 (as opposed to the whole-circle obtain in Cohort 1) was employed to assess the translational potential of our diagnostic strategy under real-world conditions, it may be prone to sampling bias. Fifth, MRE reproducibility findings across different scanners and institutions require further validation. Future multi-center studies with standardized MRE protocols, and automated postprocessing tools are needed to confirm these findings. Additionally, expanding this approach to other regions like the colorectum could elucidate its broader applicability.

In conclusion, intestinal fibrosis exhibits spatial heterogeneity. The subregionalization strategy introduced in this study addressed the diagnostic challenges posed by this heterogeneity. By separately evaluating mesenteric and antimesenteric borders, we achieved improved diagnostic accuracy compared to whole-circle assessment. Precise stratification of fibrosis severity facilitates tailored therapeutic decision-making, including surgical resection for advanced fibrosis *versus* anti-inflammatory therapy for milder cases, while identifying high-risk patients with disease progression.

## Supplementary information

**Additional file 1**: **Supplementary material 1**: MRI protocol and scan parameters. **Supplementary material 2**: Development of MCFI on MRE and its feasibility assessment. **Supplementary material 3**: Interpretation and measurement of the six MRE parameters. **Supplementary material 4**: The workflow of specimen collection in the two surgical cohorts. **Supplementary material 5**: Logistics models developed using multivariable MRE parameters for distinguishing non-mild from moderate-severe intestinal fibrosis in the mesenteric border, antimesenteric border, and whole-circle regions. **Supplementary Table S1**: Histologic scores for intestinal fibrosis. **Supplementary Table S2**: The distribution of histological fibrotic severity in three intestinal regions in Surgical Cohort 1. **Supplementary Table S3**: The differences of α-SMA+ cell information and the thickness of the muscularis propria among three regions in Surgical Cohort 1. **Supplementary Table S4**: Correlations of MRE parameters with histological fibrosis scores in mesenteric and antimesenteric border regions in surgical Cohort 2. **Supplementary Table S5**: 95% confidence interval of AUROCs for univariable MRE models in the three regions in Surgical Cohort 1. **Supplementary Table S6**: Delong test for comparisons in AUROCs between univariable and multivariable MRE models in the mesenteric border region in Surgical Cohort 1. **Supplementary Table S7**: Delong test for comparisons in AUROCs between univariable and multivariable MRE models in the antimesenteric region in Surgical Cohort 1. **Supplementary Table S8**: Delong test for comparisons in AUROCs between univariable and multivariable MRE models in the whole-circle region in Surgical Cohort 1. **Supplementary Table S9**: Delong test between each multivariable model in the mesenteric border, antimesenteric border, and whole-circle region (none-mild fibrosis *versus* moderate-severe fibrosis lesions) in Surgical Cohort 1. **Supplementary Fig. S1**: The quantitative evaluation of collagen in the mesenteric border, antimesenteric border, and whole-circle bowel walls. **Supplementary Fig. S2**: The measurement of α-SMA+ area fractions and staining intensity at the submucosa in the mesenteric border, antimesenteric border, and whole-circle bowel walls. **Supplementary Fig. S3**: The measurement of the thickness of the muscularis propria in the mesenteric border, antimesenteric border, and whole-circle bowel walls. **Supplementary Fig. S4**: SHAP plots illustrate the contribution of each MRE parameter to the model's prediction in the (**a**) mesenteric border, (**b**) antimesenteric border, and (**c**) whole-circle regions. **Supplementary Fig. S5**: Workflow and input/output structure of the APP-based calculator for intestinal fibrosis.

## Data Availability

The data that support the findings of this study are available from the corresponding author, Xuehua Li (email: lxueh@mail.sysu.edu.cn.), upon reasonable request. The data are not publicly available since this could compromise the privacy of research participants.

## Abbreviations

3D: Three-dimensional
ADC: Apparent diffusion coefficient
AUROC: Area under the receiver operating characteristic curve
CD: Crohn disease
DWI: Diffusion-weighted imaging
IQR: Interquartile range
MCFI: Mesenteric creeping fat index
MRE: Magnetic resonance enterography
MTR: Magnetization transfer ratio
SMA: Smooth muscle actin
T2WI: T2-weighted imaging
