# Supplementary material for: Mesenteric and antimesenteric border subregionalization using MR enterography: advancing fibrosis evaluation in Crohn disease
Source: Eur Radiol Exp. 2026 Mar 30;10:36. doi: 10.1186/s41747-026-00700-7 (PMC13035955; doi:10.1186/s41747-026-00700-7)
Supplement: Supplementary file 1 — Additional file 1: Supplementary material 1: MRI protocol and scan parameters. Supplementary material 2: Development of MCFI on MRE and its feasibility assessment. Supplementary material 3: Interpretation and measurement of the six MRE parameters. Supplementary material 4: The workflow of specimen collection in the two surgical cohorts. Supplementary material 5: Logistics models developed using multivariable MRE parameters for distinguishing non-mild from moderate-severe intestinal fibrosis in the mesenteric border, antimesenteric border, and whole-circle regions. Supplementary Table S1: Histologic scores for intestinal fibrosis. Supplementary Table S2: The distribution of histological fibrotic severity in three intestinal regions in Surgical Cohort 1. Supplementary Table S3: The differences of α-SMA+ cell information and the thickness of the muscularis propria among three regions in Surgical Cohort 1. Supplementary Table S4: Correlations of MRE parameters with histological fibrosis scores in mesenteric and antimesenteric border regions in surgical Cohort 2. Supplementary Table S5: 95% confidence interval of AUROCs for univariable MRE models in the three regions in Surgical Cohort 1. Supplementary Table S6: Delong test for comparisons in AUROCs between univariable and multivariable MRE models in the mesenteric border region in Surgical Cohort 1. Supplementary Table S7: Delong test for comparisons in AUROCs between univariable and multivariable MRE models in the antimesenteric region in Surgical Cohort 1. Supplementary Table S8: Delong test for comparisons in AUROCs between univariable and multivariable MRE models in the whole-circle region in Surgical Cohort 1. Supplementary Table S9: Delong test between each multivariable model in the mesenteric border, antimesenteric border, and whole-circle region (none-mild fibrosis versus moderate-severe fibrosis lesions) in Surgical Cohort 1. Supplementary Fig. S1: The quantitative evaluation of collagen in the mesenteric bord [file 41747_2026_700_MOESM1_ESM.pdf]

Mesenteric and antimesenteric border subregionalization using MR enterography: advancing fibrosis evaluation in Crohn disease

ELECTRONIC SUPPLEMENTARY MATERIAL

Contents

Supplementary materials 1

Reader's Guide: Navigating the Supplementary Materials 3

Supplementary material 1: MRI protocol and scan parameters 5

Supplementary material 2: Development of MCFI on MRE and its feasibility assessment 7

Supplementary material 3: Interpretation and measurement of the six MRE parameters 10

Supplementary material 4: The workflow of specimen collection in the two surgical cohorts 14

Supplementary material 5: Logistics models developed using multivariable MRE parameters for distinguishing none-mild from moderate-severe intestinal fibrosis in the mesenteric border, antimesenteric border, and whole-circle regions 17

Mesenteric border region 17

Antimesenteric border region 17

Whole-circle region 18

Supplementary Tables 19

Supplementary Table S1: Histologic scores for intestinal fibrosis 19

Supplementary Table S2: The distribution of histological fibrotic severity in three intestinal regions in surgical Cohort 1 20

Eur Radiol Exp (2026) Wu LY, Lin JJ, Zheng WK, et al.

Supplementary Table S3: The differences of α-SMA⁺ cell information and the thickness of the muscularis propria among three regions in surgical Cohort 1 21
Supplementary Table S4: Correlations of MRE parameters with histological fibrosis scores in mesenteric and antimesenteric border regions in surgical Cohort 2 21
Supplementary Table S5: 95% confidence interval of AUROCs for univariable MRE models in the three regions in surgical Cohort 1 22
Supplementary Table S6: Delong test for comparisons in AUROCs between univariable and multivariable MRE models in mesenteric border region in surgical Cohort 1 23
Supplementary Table S7: Delong test for comparisons in AUROCs between univariable and multivariable MRE models in the antimesenteric region in surgical Cohort 1 24
Supplementary Table S8: Delong test for comparisons in AUROCs between univariable and multivariable MRE models in the whole-circle region in surgical Cohort 1 25
Supplementary Table S9: Delong test between each multivariable model in the mesenteric border, antimesenteric border, and whole-circle region (none-mild fibrosis vs. moderate-severe fibrosis lesions) in surgical Cohort 1... 26
Supplementary Figures... 27
Supplementary Fig. S1 The quantitative evaluation of collagen in the mesenteric border, antimesenteric border, and whole-circle bowel walls. 27
Supplementary Fig. S2 The measurement of α-SMA⁺ area fractions and staining intensity at submucosa in the mesenteric border, antimesenteric border, and whole-circle bowel walls. 29
Supplementary Fig. S3 The measurement of thickness of the muscularis propria in the mesenteric border, antimesenteric border, and whole-circle bowel walls. 31
Supplementary Fig. S4 SHAP plots illustrate the contribution of each MRE parameter to the model's prediction in the (a) mesenteric border, (b) antimesenteric border, and (c) whole-circle regions. 33
Supplementary Fig. S5 Workflow and input/output structure of the APP-based calculator for intestinal fibrosis. 37
Appendix: Overview of All Scoring Systems and Parameters.................................. 39
Reference... 42

Reader's Guide: Navigating the Supplementary Materials

To facilitate a clear understanding of the extensive data presented in this study, this guide organizes the Supplementary Materials according to the main research workflow. The materials are categorized into two primary sections: 1) Methodological Foundations and 2) Extended Results and Validation. Readers interested in the technical implementation can focus on the first section, while those seeking deeper analytical insights may proceed directly to the second.

Part I: Methodological Foundations

This section provides comprehensive details on the experimental methods, imaging protocols, and technical validation procedures.

Supplementary Material 1: MRI Protocol and Scan Parameters

Details the standardized MRI sequences and parameters used for both *in vivo* patient scans and *ex vivo* specimen imaging, ensuring reproducibility.

Supplementary Material 2: Development of MCFI on MRE and Its Feasibility Assessment

Describes the novel application and validation of the MCFI on MRE, including quality assessment and cross-modality comparison with CTE.

Supplementary Material 3: Interpretation and Measurement of the Six MRE Parameters

Offers a step-by-step visual and descriptive guide on how the six MRE parameters were qualitatively scored and quantitatively measured within the defined subregions.

Supplementary Material 4: The Workflow of Specimen Collection in the Two Surgical Cohorts

Offers a step-by-step visual and descriptive guide on how the six MRE parameters were qualitatively scored and quantitatively measured within the defined subregions.

Appendix: Overview of All Scoring Systems and Parameters

Serves as a quick-reference table summarizing all scoring systems and quantitative parameters used throughout the study.

Part II: Extended Results and Validation

This section contains additional data that robustly support the main findings, including extended pathological analyses, detailed model performance, and clinical outcome validations.

Supplementary Material 5: Logistics Models for Multivariable MRE Analysis

Presents the complete mathematical formulas for the five multivariable models developed for each intestinal region (mesenteric border, antimesenteric border, whole-circle), enabling external validation and application.

Supplementary Tables S2-S4: Additional Evidence for Fibrosis Heterogeneity and MRE Correlations

Provides patient-level distribution of fibrotic severity (Supplementary Table 2), additional pathological metrics like α-SMA (Supplementary Table 3), and corroborating correlations between MRE parameters and histology from the validation cohort (Supplementary Table 4).

Supplementary Tables S5-S9: Comprehensive Diagnostic Performance Analysis

Contains full details on model performance, including confidence intervals for all univariable models (Supplementary Table 5) and statistical comparisons (DeLong's test) between all developed models (Supplementary Tables 6-9), demonstrating the incremental value of multivariable analysis and subregionalization.

Supplementary Fig. S1-S3: Detailed Pathological Quantification Methods

Illustrates the meticulous image analysis workflows used for quantifying collagen, α-SMA$^+$ cells, and muscularis propria thickness, validating the pathological basis of fibrosis heterogeneity.

Supplementary Fig. S4: SHAP Analysis for Model Interpretation

Uses SHAP to visually interpret the optimal models, identifying the most influential MRE parameters driving fibrosis predictions in each subregion.

Supplementary Fig. S5: APP-Based Calculator Workflow

Demonstrates the user interface and functionality of the open-access APP that implements our optimal models, highlighting the clinical translatability of our diagnostic strategy.

Supplementary material 1: MRI protocol and scan parameters

After bowel preparation, 1600 to 2000ml of a 2.5% mannitol solution was administered for participants within one hour prior to the MR enterography (MRE) examination. An intramuscular injection of 10mg anisodamine (6-542) was administered in the gluteus ten minutes before the MRE examination to inhibit gastrointestinal peristalsis.

In both hospitals, MRE was conducted using a 3-T magnetic resonance system (MAGNETOM Prisma - Tim and Dot System; Siemens Healthineers, Erlangen, Germany), along with a multichannel phased-array coil. The multi-parameter MRE for participants included T2-weighted imaging (T2WI), diffusion-weighted imaging (DWI), magnetization transfer imaging (MTI), pre-/post-enhanced T1-mapping, and pre-/post-enhanced T1-weighted imaging (T1WI). The acquisition of post-enhanced T1-mapping was performed at approximately 5 minutes after contrast administration; while the acquisition of post-enhanced T1WI was conducted at 28 s (coronal), 70 s (coronal), 90 s (coronal), 3 min (axial), and 3.5 min (coronal) after contrast administration.

Additionally, the targeted intestinal specimens that had been removed from participants in surgical cohort 1were immediately scanned again using 3D-SPACE-T2WI after surgery, to identify their morphological characteristics.

The scan parameters for participants in the both hospitals or intestinal specimens are detailed in the following table.

MRI scan sequences and parameter*s*

Parameters	T2-weighted HASTE	MT GRE	Diffusion-weighted SE-EPI	T1 Mapping-vibe	T1 weighted-vibe	T2-weighted-3D SPACE†
Orientation	2D axial	2D axial	2D axial	3D axial	3D axial/3D coronal	3D axial
Field of View (mm)	380x297	350x208	380x283	380x306	380x306/450x394	199x199
Acquisition matrix	320x243	256x205	134x134	320x240	320x240/320x256	384x384
Flip angle (degrees)	160	30	90.0	3.0, 15.0	9.0/10.5	120.0
Section thickness (mm)	4.0	4.0	4.0	3.0	3.0/2.0	0.5
Slice distance	0.8	0.8	0.8	no gap	no gap/no gap	no gap

No. of sections	45	10	45	72	72/88	100
Echo time (msec)	81	2.81	47	1.3	1.3/1.35, 2.58	132.0
Repetition time (msec)	1000	223	5400	3.31	3.31/4.28	1000.0
Averages	1	1	1, 2, 2	1	1/1	2
Respiratory control	Breath hold	Breath hold	Free-breathing	Breath hold	Breath hold/Breath hold	None
b factors (sec/mm^2)	None	None	50, 400, 800	None	None/None	None
Diffusion Mode	None	None	3-Scan Trace	None	None/None	None
Diffusion Scheme	None	None	Monopolar	None	None/None	None
Acquisition time (sec)	57.0	26.0	105.0	34.0	17.0/23.0	450.0
Concatenations	3	2	1	2	1/1	1
Fat saturation	None	None	SPAIR	None	SPAIR/None	None
Fat saturation mode	None	None	Strong	None	None/Dixon	None
MTC	None	on/off	None	None	None/None	None
Frequency offset (kHz)	None	1.2	None	None	None/None	None
Frequency offset duration (μsec)	None	9984	None	None	None/None	None
Frequency offset effective flip angle	None	500	None	None	None/None	None
Frequency offset bandwidth (Hz)	None	192	None	None	None/None	None

Note-*HASTE* half-Fourier acquisition single-shot turbo spin-echo; *GRE* gradient echo; *SE-EPI* spin-echo echo-planar imaging; *vibe* volumetric interpolated breath-hold examination; *3D* three-dimensional; *SPACE* Sampling Perfection with Application optimized Contrast using different flip angle Evolution.

†The MRI sequence that used to scan intestinal specimens *ex vivo*.

Supplementary material 2: Development of MCFI on MRE and its feasibility assessment

Development of MCFI on MRE

Mesenteric creeping fat index (MCFI) is an anatomical imaging index developed based on mesenteric vessels for characterizing creeping fat wrapping around the diseased gut, which is first reported in a study [1] on CT enterography (CTE) for Crohn disease (CD) and has recently used in another CD study on MRE (doi:10.3969/j.issn.1006-5725.2024.05.013). The technical details presented in these two published studies indicate that the reconstruction of MCFI does not necessarily require a fixed image orientation or an enhanced phase, as long as the mesenteric vessels surrounding the target intestine can be successfully reconstructed. Therefore, two radiologists (L.Y.W and Y.D.W, with two and ten years of experience in abdominal imaging respectively and no access to specimen or pathological information) used axial post-enhanced T1WI at 3 minutes post-contrast administration in our study to develop MCFI for most of CD patients, primarily due to its convenience in reconstructing this index in the terminal ileum. Specifically, MCFI was constructed by acquiring an axial view of the targeted intestine with its adjacent mesenteric vessels obtained by orienting the slice perpendicular to its longitudinal axis, using multiplanar reconstruction. The circumference of the intestine is subdivided into 8 equal zones, and a score of 1 is applied for each zone that overlaps with mesenteric vessels. As a result, MCFI is scored on a scale from one to eight (**Figure 3**).

Reconstruction quality assessment of MCFI on MRE

The reconstruction quality of MCFI on MRE in the three cohorts was assessed on a scale ranging from 0 to 2 by the two radiologists.

A score of 0 indicated low quality, with blurred mesenteric vessels and an indistinguishable mesenteric border region.

A score of 1 denoted moderate quality, where the mesenteric vessels were visible and the mesenteric border region could be identified.

A score of 2 represented high quality, with clearly visible mesenteric vessels and accurate identification of the mesenteric border region.

The representative cases are displayed below:

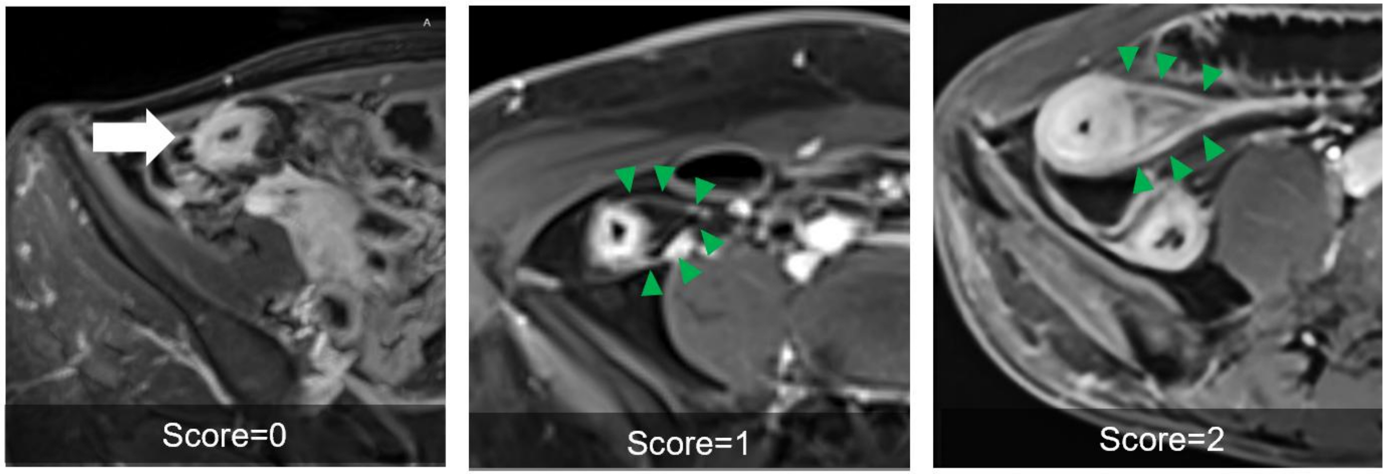

■ Reconstructive quality assessment and scoring scheme for MCFI on MRE in the terminal ileum

Comparison of MCFI reconstruction on MRE and CTE

Due to MCFI is initially reported in CT studies, to further validate the feasibility of MCFI development on MRE, we additionally conducted a comparative analysis in MCFI between CTE images and MRE images in two subgroups where each patient underwent both imaging modalities within a three-month interval. The first subgroup consisted of six patients with CD included in the surgical cohort 1, while the second cohort comprised 20 patients with terminal ileum CD admitted to our hospital (Yuexiu) from July 2023 to July 2024.

In subgroup 1, the median [IQR] values of the MCFI on MRE and the MCFI on CTE were 3.5 [2.92, 4.08] and 3.5 [2.00, 4.17], respectively. The MCFI on MRE was significantly correlated with MCFI on CTE (r=0.96, p < 0.001). In subgroup 2, the median [IQR] values of MCFI on MRE and MCFI on CTE were both 3 [2, 4]. The MCFI on MRE was also significantly correlated with MCFI on CTE (r=0.89, p < 0.001). These results provided strong evidence supporting the feasibility of using MRE to develop MCFI. Two representative cases from subgroups 1 and 2 respectively also demonstrated a consistent wrapping degree between the MCFI on MRE and the MCFI on CTE in the same patient with terminal ileum CD.

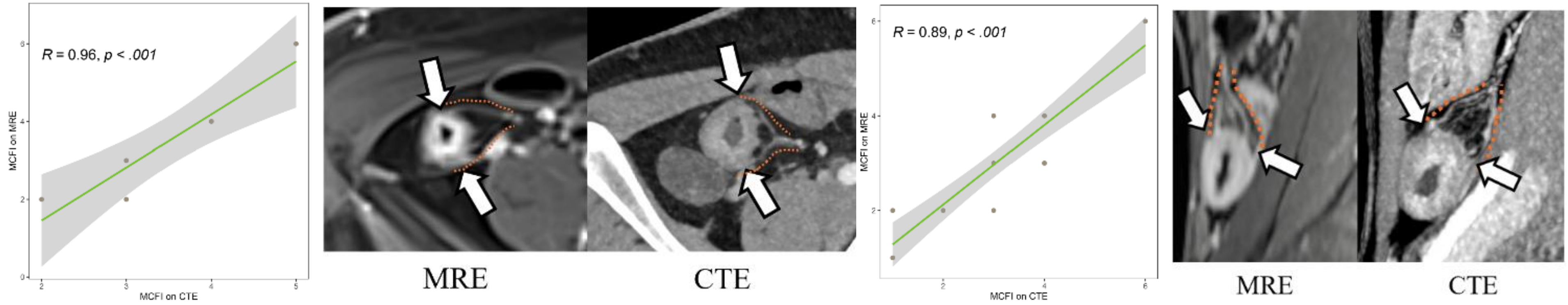

A B C D

- The MRE-based MCFI and the CTE-based MCFI show a significantly positive correlation in identical patients from subgroup 1 (*n* =6; figure A). A representative case (figure B; 32-year-old, male) from subgroup 1 demonstrates a consistent wrapping degree between MCFI on MRE (axial post-enhanced T1WI at 3 minutes post-contrast administration) and that observed on CTE (axial venous-phase image) in the same patient (both MCFI=4). Similarly, there is a significantly positive correlation between the MRE-based MCFI and the CTE-based MCFI in identical patients from subgroup 2 (*n* =20; figure C). Another representative case (figure D; 27-year-old, male) from subgroup 2 demonstrates a consistent wrapping degree between MCFI on MRE (sagittal image constructed by axial post-enhanced T1WI at 3 minutes post-contrast administration) and that observed on CTE (sagittal image constructed by axial arterial-phase image) in the same patient (both MCFI=2). The time interval between CTE and MRE for the two patients are both two months. The orange dotted lines and white arrows depict the outer edge of the mesenteric vessels that wrap around the diseased intestine.

Supplementary material 3: Interpretation and measurement of the six MRE parameters

Workflow for slice selection and regional MRE parameter measurement - The procedure for selecting the axial level and measuring the six MRE parameters followed a standardized protocol to ensure consistency and precise histology correlation. The following steps detail the process for the mesenteric border region, with the same methodological principles of slice selection and ROI application being applied to the antimesenteric border region and whole-circle region. The coronal post-enhanced T1WI slice that traversed the narrowest cross-section of the target intestinal segment (red dotted line) was identified for all subsequent analysis (Figure a). This coronal level was then used to pinpoint the matching axial slice on the 3-minute post-contrast T1WI, which exhibited the most severe luminal narrowing and wall thickening for cross-sectional analysis. On this axial index slice, the mesenteric border of the intestinal wall (green shaded area) was delineated based on the MCFI (Figure b). All six parameters were then assessed within the defined regions. Qualitative parameters (T2WI and DWI signals) were collectively evaluated by three blinded radiologists (X.H.L, S.T.F, and R.N.Z; 8–20 years of experience). Quantitative parameters (normalized MTR, ADC, pre- and post-enhanced T1 values) were measured by radiologist X.H.L, who placed two to three ROIs within the mesenteric border on parametric maps with common approval from the others, carefully avoiding artifacts and luminal contents; the average of these measurements was used for statistical analysis (Figure c).

This consistent approach was applied with cohort-specific focus: In Cohort 1, axial MRE slices corresponding to the 3D-printed target intestinal segment were selected at 4.8 mm intervals, aligned with the T2-weighted HASTE sequence parameters. In Cohort 2, analysis was focused on the single axial slice corresponding to the narrowest luminal cross-section, which matched the level from which surgical specimens were obtained. In the follow-up cohort, assessment was performed at the baseline axial slice exhibiting the most pronounced intestinal wall thickening.

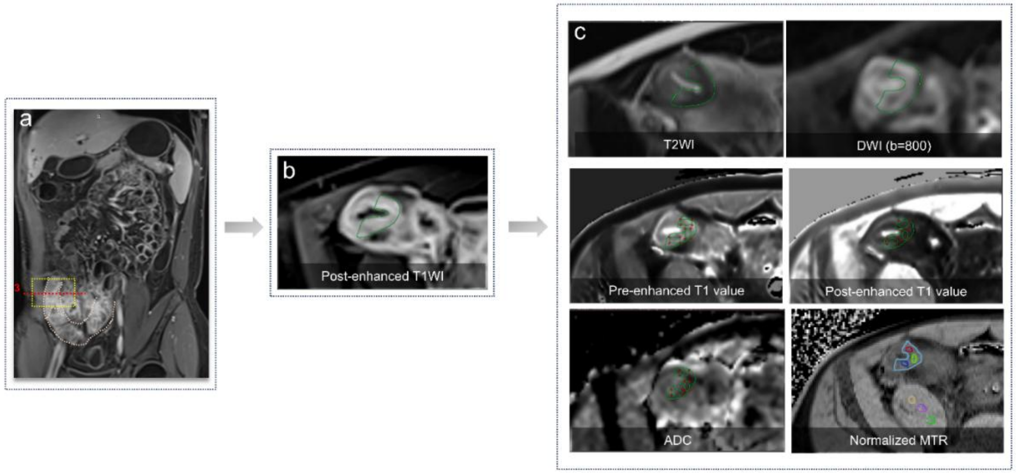

■ Illustration of slice selection and ROI measurement

T2-weighted imaging (T2WI) signal - The T2WI signal intensity of bowel wall was evaluated using a 3-point scale: isointensity compared to adjacent normal bowel wall (score 0), slightly increased intensity with dark grey appearance (score 1), significantly increased intensity with white gray or white appearance (score 2)[2]. The scoring diagram is depicted below. Assessment of T2WI signal intensity was performed using RadiAnt DICOM Viewer (version 2021.2; Medixant; Promienista 2560-288 Poznań Poland). Moreover, an independent evaluation of the T2WI signal was conducted by the radiologist (X.H.L) three months after the team's imaging assessment. Subsequently, an analysis of inter-observer consistency in T2WI signal between these two evaluations was performed. The intraclass correlation coefficient (ICC) values for inter-observer consistency in T2WI signal are 0.932, 0.855, and 0.843 in the surgical cohort 1, surgical cohort 2, and follow-up cohort, respectively. The complete results across all cohorts are summarized in the table below for clarity.

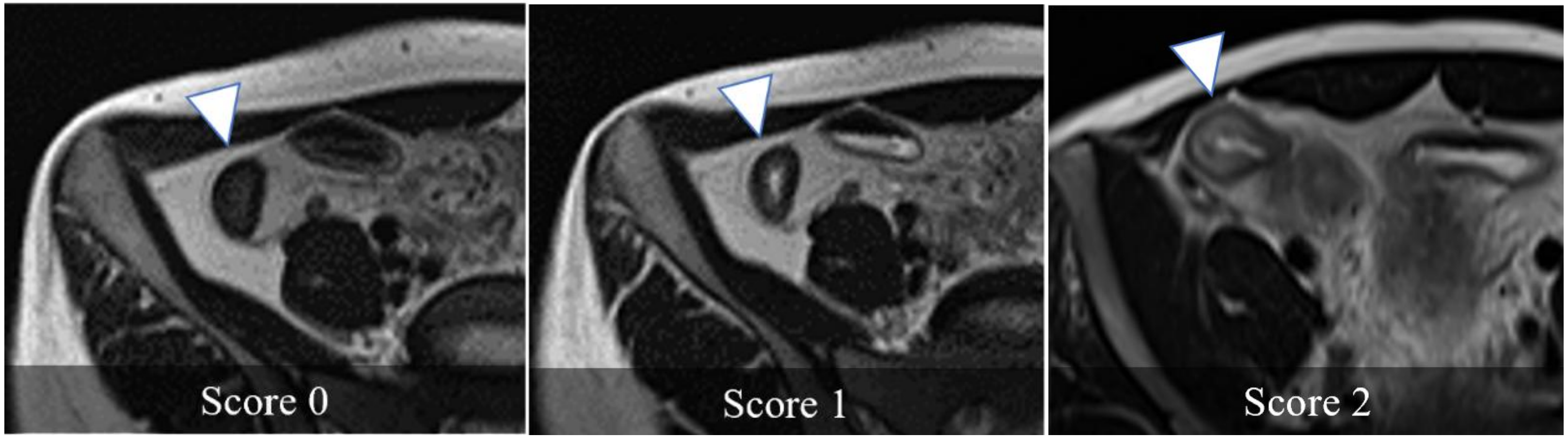

■ The T2WI signal alterations of the terminal ileum and their corresponding scoring scheme

Table. Interobserver Agreement (ICC) for T2WI Signal Intensity by Cohort and Intestinal Region

Intestinal Region	Surgical Cohort 1	Surgical Cohort 2	Follow-up Cohort
Mesenteric Border	0.882	0.833	0.870
Antimesenteric Border	0.924	0.871	0.826
Whole-Circle	0.983	-	0.807
Overall	0.932	0.855	0.843

Diffusion-weighted imaging (DWI) signal – DWI signal intensity of bowel wall was evaluated using a 3-point scale modified from a previous study [3]: isointensity (score 0), slightly increased intensity (score 1), significantly increased intensity (score 2), mainly compared to normal bowel wall. The scoring diagram is depicted below. This assessment was also conducted using the RadiAntViewer. Moreover, an independent evaluation of the DWI scores was conducted by the radiologist (X.H.L) three months after the team's imaging assessment. Subsequently, an analysis of inter-observer consistency in DWI scores between these two evaluations was performed. The ICC values for inter-observer consistency in DWI signal are 0.941, 0.842, 0.828 in the surgical cohort 1, surgical cohort 2, and follow-up cohort respectively. The complete results across all cohorts are summarized in the table below for clarity.

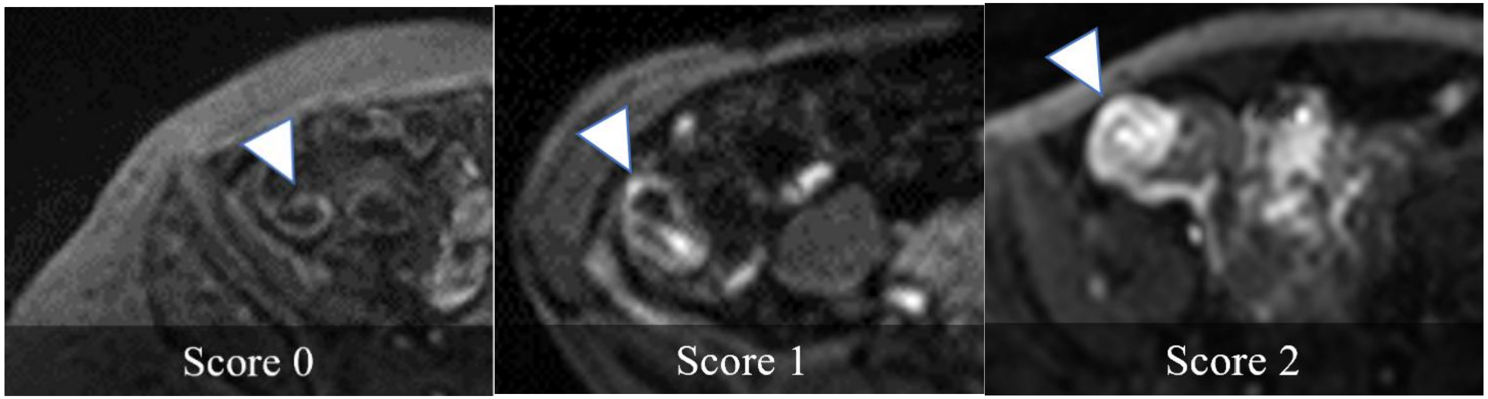

■ The DWI signal alterations of the terminal ileum and their corresponding scoring scheme

Table: Interobserver Agreement (ICC) for DWI Signal Intensity by Cohort and Intestinal Region

Intestinal Region	Surgical Cohort 1	Surgical Cohort 2	Follow-up Cohort
Mesenteric Border	0.946	0.881	0.859
Antimesenteric Border	0.951	0.806	0.795
Whole-Circle	0.928	-	0.796
Overall	0.941	0.787	0.828

Normalized magnetization transfer ratio (MTR) - MTR was performed using an in-house Matlab script, as previously described in our prior study [4]. MTR was calculated as $\mathrm{MTR} = \frac{M_0 - M_{Sat}}{M_0} \times 100\%$, where M_{sat} and M_0 represent the signal intensity obtained with and without the application of off-resonance presaturation pulse, respectively. To minimize individual differences, the MTR of the bowel wall was divided by the MTR of the muscle, using the following formula: $\text{Normalized MTR} = \frac{\text{MTR bowel wall}}{\text{MTR psoas muscle}}$.

The apparent diffusion coefficient (ADC) – The ADC maps were automatically reconstructed by using three b values of 50 s/mm², 400 s/mm², and 800 s/mm². We measured ADC values of bowel walls using the RadiAnt DICOM Viewer.

T1 value - Quantitative T1 maps were automatically reconstructed on a voxel-by-voxel basis after data acquisition using the MapIt processing tool (MapIt software, Siemens Healthineers, Erlangen, Germany). Pre-/post-enhanced T1 values were measured using the RadiAnt DICOM Viewer.

Supplementary material 4: The workflow of specimen collection in the two surgical cohorts

S4.1 Overview

This section delineates the workflow for specimen collection and coregistration in the two surgical cohorts. Cohort 1 utilized a customized 3D-printing protocol for precise layer-by-layer coregistration, while Cohort 2 followed a standard clinical procedure.

S4.2 In surgical cohort 1: Detailed Protocol with 3D-Printing Guided Coregistration

The following comprehensive technical details, referenced from the Methods section, elaborate on the 3D-printing process used in surgical cohort 1:

Three radiologists (X.H.L, S.T.F, and R.N.Z) collaborated with a senior gastrointestinal surgeon (Z.H.C) to identify the resected extent of terminal ileum (from ileocecal valve to its proximal end). Important anatomical landmarks, such as ileocecal valve, narrowest layer, and length of the resected intestine, were accurately determined on post-enhanced T1WI. 3D printing technology and lesion characteristics were used to achieve longitudinal coregistration between MRE images and both intestinal specimens and pathological sections, as previously described in studies on other diseases [5, 6]. The sequential steps were as follows:

A. 3D delineation of the volume of interest of the targeted intestinal segment: We first imported the post-enhanced T1WI images into VitreaWorkStation (version 6.5.5; Vital Images, Inc; 5850 Opus Parkway, Suite 300; Minnetonka, MN, US; 55343). Subsequently, based on the information obtained from multisequence MRE interpreted by both radiologists and surgeon to determine the targeted intestinal segment, we carefully delineated a 3D volume of interest along the contour of this specific intestinal segment on these post-enhanced T1WI images and saved it as STL data.

B. Design of a 3D-printing mold with slots for the targeted intestine segment: The aforementioned 3D data (STL) were then imported into AutoDesk Fusion360 (version 2.0.16490; Autodesk Asia Pte. Ltd.3 Fusionopolis Way#10-21 Symbiosis Singapore 138633) to reconstruct the 3D model of the targeted intestine segment, followed by designing a 3D-printing mold according to this model. The size and shape of the cavity within the 3D-printing mold used to contain the surgically resected specimen were consistent with those of the targeted intestinal segment depicted on T1W images. The positioning and quantity of the cutting slots in the 3D-printing molds were determined according to the scanning parameters of the axial T2-weighted HASTE images. Specifically, the width of every slot was 2 mm. The spacing between the two adjacent slots, defined as the vertical distance from the lower edge of the previous slot to the upper edge of the next slot, measured 2.8 mm. The distance between the center lines of the two adjacent slots was therefore 4.8 mm

(2/2 mm+2.8 mm+2/2 mm=4.8 mm), which aligned with the corresponding distance between the center lines of the T2WI-Haste images (with a section thickness of 4 mm and a slice distance of 0.8 mm, as specified in Supplementary Material 1). The 3D printed mold had an "H" identifier (as depicted below), indicating that this side represents the distal side of the corresponding intestinal specimen. The first slot in the 3D printing mold usually corresponded to the distal end of the target intestine, which was located near or at the level of the ileocecal valve, while the last slot typically corresponded to the proximal end of the targeted intestine. The slots were evenly spaced, and one of the middle slots accurately identified the central level of the narrowest part of the target intestine and clearly marked it on the 3D printing mold.

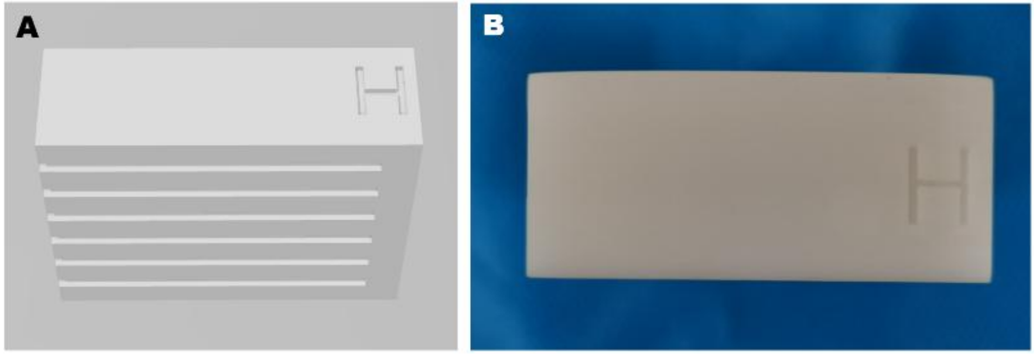

- The electronically designed mold diagram (A) is labelled with "H" and subsequently transformed into a tangible mold (B) using 3D-printing technology, indicating that this side represents the distal side of the corresponding intestinal specimen.

C. 3D printing to transform digital molds into tangible objects: The 3D digital model was transformed into a tangible mold using 3D printing equipment from the Bambu Lab (Bambu Lab version X1E; Bambu Lab, Guoxin Investment Building, Nanshan District, Shenzhen, Guangdong Province).

D. Surgical procedures and ex vivo MRI scan of the targeted intestinal segment: Based on the information obtained from multisequence MRE interpreted by both radiologists and surgeon to determine the targeted resected intestinal segment, the surgeon carefully resected the terminal ileum and its adjacent creeping fat and employed a suture to tie knots on the creeping fat next to the ileocecal valve for marking it as the distal end of the terminal ileum. The intestinal specimen was carefully placed in a refrigerated specimen transfer box without fixation or any solution and transported to the MRI scanning room. The intestinal specimen was immediately subjected to T2W-3D-SPACE *ex vivo* scan (**Supplementary Material 1**) by a designated radiologist to obtain additional anatomical information with the corresponding surface identification, including the distal end, the narrowest layer, and the proximal end of the target bowel segment, for subsequent coregistration between *in vivo* and *ex vivo*.

E. Repositioning the intestinal specimen into a 3D-printing mold: The intestinal specimen was restored to its original configuration based on the orientation of the MRE images (particularly T2WI-HASTE) and placed within the 3D printed mold using previously obtained positioning marks by the designated radiologist. Specifically, we aligned the upper margin of the intestinal specimen with the first slot, matched the narrowest luminal level of the specimen with the marked slot (corresponding to the narrowest luminal level of the intestine on *in vivo* MRE images), and aligned the lower margin of the specimen with the last slot. By aligning these three levels, we were able to achieve longitudinal coregistration between MRE images and specimens.

F. Sectioning the specimen along the designated slots: The intestinal specimen was dissected along the designated slots to obtain axial whole-circle sections of the intestine by the designated radiologist. Each section had a thickness of approximately 4.8 mm.

G. Pathological whole-circle whole-slide imaging of the intestinal wall: The intestinal sections were further sliced into 4-μm-thick subsections and subjected to Masson trichrome staining. Subsequently, the stained pathological subsections were digitally scanned to obtain electronic whole-circle whole-slide images for histopathological assessment using PANNORAMIC DESK (3DHISTECH Ltd; H-1141 Budapest, Öv u. 3, Hungary). CaseViewer (version 2.4; 3DHISTECH Ltd.; H-1141 Budapest, Öv u. 3, Hungary) was used for histological analysis.

The above steps A, B, D, E, F were conducted by the designated radiologist (J.J.L, with 10 years of abdominal imaging experience) unless otherwise specified.

S4.3 Surgical cohort 2: Standard Clinical Procedure

For Cohort 2, we followed the common procedure for specimen collection. Briefly, we relied on pre-/intra-operative observations for lesion coregistration, instead of using 3D-printing. Similarly, three radiologists collaborated with the senior gastrointestinal surgeon to identify the resected extent of terminal ileum (from ileocecal valve to its proximal end). Important anatomical landmarks, such as ileocecal valve, narrowest layer, and length of the resected intestine, were accurately determined on preoperative MRE and during intraoperation to achieve accurate region-by-region coregistration between MRE images and specimens. After performing coregistration between the modalities, two small pieces of full-thickness tissues were obtained from both mesenteric and antimesenteric border regions at the same axial level of the gut. The other analysis procedures utilized in this cohort were identical to those in surgical cohort 1.

Supplementary material 5: Logistics models developed using multivariable MRE parameters for distinguishing none-mild from moderate-severe intestinal fibrosis in the mesenteric border, antimesenteric border, and whole-circle regions

Mesenteric border region

$$Y_1 = -0.721 \times T2WI\ signal + 3.284 \times DWI\ signal - 2.633$$

$$Model\ 1 = \frac{1}{1 + e^{(-Y_1)}}$$

$$Y_2 = -0.004 \times ADC - 0.788 \times T2WI\ signal + 2.268 \times DWI\ signal + 4.704$$

$$Model\ 2 = \frac{1}{1 + e^{(-Y_2)}}$$

$$Y_3 = -0.003 \times ADC + 11.188 \times Normalized\ MTR - 0.311 \times T2WI\ signal + 1.251 \times DWI\ signal - 3.462$$

$$Model\ 3 = \frac{1}{1 + e^{(-Y_3)}}$$

$$Y_4 = -0.003 \times ADC + 11.536 \times Normalized\ MTR - 0.004 \times postenhanced\ T1\ value - 0.092 \times T2WI\ signal + 1.069 \times DWI\ signal - 1.564$$

$$Model\ 4 = \frac{1}{1 + e^{(-Y_4)}}$$

$$Y_5 = -0.003 \times ADC + 11.536 \times Normalized\ MTR - 0.001 \times preenhanced\ T1\ value - 0.003 \times postenhanced\ T1\ value + 0.099 \times T2WI\ signal + 0.811 \times DWI\ signal + 0.546$$

$$Model\ 5 = \frac{1}{1 + e^{(-Y_5)}}$$

Antimesenteric border region

$$Y_1 = 0.096 \times T2WI\ signal + 1.706 \times DWI\ signal - 3.204$$

$$Model\ 1 = \frac{1}{1 + e^{(-Y_1)}}$$

$$Y_2 = -1.626 \times 10^{-3} \times ADC - 0.123 \times T2WI\ signal + 1.408 \times DWI\ signal - 0.165$$

$$Model\ 2 = \frac{1}{1 + e^{(-Y_2)}}$$

$$Y_3 = -9.424 \times 10^{-5} \times ADC + 12.789 \times Normalized\ MTR - 0.281 \times T2WI\ signal + 1.176 \times DWI\ signal - 10.575$$

$$Model\ 3 = \frac{1}{1 + e^{(-Y_3)}}$$

$$Y_4 = 2.795 \times 10^{-5} \times DWI + 12.727 \times Normalized\ MTR - 2.494 \times 10^{-3} \times postenhanced\ T1\ value - 0.193 \times T2WI\ signal + 0.927 \times DWI\ signal - 9.242$$

$$Model\ 4 = \frac{1}{1+e^{(-Y_4)}}$$

$$Y_5 = 1.717 \times 10^{-4} \times ADC + 13.687 \times Normalized\ MTR - 2.421 \times 10^{-3} \times preenhanced\ T1\ value - 2.315 \times 10^{-3} \times postenhanced\ T1\ value + 0.322 \times T2WI\ signal + 0.659 \times DWI\ signal - 6.620$$

$$Model\ 5 = \frac{1}{1+e^{(-Y_5)}}$$

Whole-circle region

$$Y_1 = -0.521 \times T2WI\ signal + 2.735 \times DWI\ signal - 4.482$$

$$Model\ 1 = \frac{1}{1+e^{(-Y_1)}}$$

$$Y_2 = -9.931 \times 10^{-5} \times ADC - 0.497 \times T2WI\ signal + 2.747 \times DWI\ signal - 4.672$$

$$Model\ 2 = \frac{1}{1+e^{(-Y_2)}}$$

$$Y_3 = 5.254 \times 10^{-4} \times ADC + 3.528 \times Normalized\ MTR - 0.622 \times T2WI\ signal + 2.594 \times DWI\ signal - 7.263$$

$$Model\ 3 = \frac{1}{1+e^{(-Y_3)}}$$

$$Y_4 = 5.807 \times 10^{-4} \times DWI + 3.417 \times Normalized\ MTR - 1.626 \times 10^{-3} \times postenhanced\ T1\ value - 0.623 \times T2WI\ signal + 2.543 \times DWI\ signal - 6.444$$

$$Model\ 4 = \frac{1}{1+e^{(-Y_4)}}$$

$$Y_5 = 4.390 \times 10^{-4} \times ADC + 2.697 \times Normalized\ MTR + 1.986 \times 10^{-3} \times preenhanced\ T1\ value - 1.865 \times 10^{-3} \times postenhanced\ T1\ value - 0.935 \times T2WI\ signal + 2.964 \times DWI\ signal - 9.193$$

$$Model\ 5 = \frac{1}{1+e^{(-Y_5)}}$$

Note: The input values of the ADC range in the hundreds or thousands, expressed in units of "$\times 10^{-6}$ mm^2/s", such as 1311$\times 10^{-6}$ mm^2/s. Other imaging parameters are directly incorporated into the formula based on their evaluation results.

Supplementary Tables

Supplementary Table S1: Histologic scores for intestinal fibrosis

Score	Fibrosis
None-mild disease	
0	No fibrosis
1	Minimal fibrosis in submucosa or subserosa
2	Increased submucosal fibrosis, septa into muscularis propria
Moderate-severe disease	
3	Septa through muscularis propria, increase in subserosal collagen
4	Significant transmural scar, marked subserosal collagen

Note: Fibrotic score in the three regions was collectively scored by two pathologists (Z.Y.Y and X.B.L, with 5 and 15 years of experience respectively and no access to clinical or imaging information).

Supplementary Table S2-S3: Additional Evidence for Spatial Heterogeneity of Fibrosis

The main text Figure 4a demonstrates significant heterogeneity in fibrosis scores and collagen fraction between regions. The data below provide the patient-level distribution and additional pathological metrics.

Supplementary Table S2: The distribution of histological fibrotic severity in three intestinal regions in surgical Cohort 1

	Mesenteric border region		Antimesenteric border region		Whole-circle region	
	None-mild fibrosis	Moderate-severe fibrosis	None-mild fibrosis	Moderate-severe fibrosis	None-mild fibrosis	Moderate-severe fibrosis
**Patient 1**	6	2	7	1	4	4
**Patient 2**	1	5	3	3	5	1
**Patient 3**	1	8	6	3	9	0
**Patient 4**	0	10	2	8	6	4
**Patient 5**	0	5	4	1	1	4
**Patient 6**	1	6	6	1	5	2
**Patient 7**	0	7	2	5	7	0

**Patient 8**	0	6	4	2	3	3
**Patient 9**	1	10	3	8	3	8
**Patient 10**	0	7	0	7	1	6
**Patient 11**	4	2	6	0	6	0
**Patient 12**	1	5	1	5	4	2
**Total**	15	73	44	44	54	34

Note: None-mild fibrosis (fibrosis scores 0-2), moderate-severe fibrosis (fibrosis 3-4). This table illustrates the consistent pattern of mesenteric-predominant fibrosis across most individuals, reinforcing the finding of spatial heterogeneity.

Supplementary Table S3: Differences in α-SMA⁺ cell metrics and muscularis propria thickness among three regions in surgical Cohort 1

	α-SMA⁺ area fractions (%)		α-SMA⁺ staining intensity (Inverse grey scale)		Thickness of the muscularis propria (μm)	
	Mean±SD	*p*-value	Mean±SD	*p*-value	Mean±SD	*p*-value
Mesenteric border	27.45±13.29	0.243	143.05±26.02	0.614	2687.08±975.67	0.716
Antimesenteric border	23.71±12.34		138.86±25.40		2564.34±917.07	
Whole-circle	25.68±12.23		141.41±24.27		2649.22±831.95	

Note: *α-SMA* alpha-smooth muscle actin; *SD* standard deviation. Analyses of α-SMA+ mesenchymal cells and muscularis propria showed a consistent trend of higher values in the mesenteric border, although these differences did not reach statistical significance in this cohort.

Supplementary Table S4: Correlations of MRE parameters with histological fibrosis scores in mesenteric and antimesenteric border regions in surgical Cohort 2

Spearman's rho		T2WI signal	DWI signal	ADC	Normalized MTR	Pre-enhanced T1 value	Post-enhanced T1 value	APP-based prediction value
Histological fibrosis scores in mesenteric border region	***r***	**0.384**	**0.264**	**-0.372**	**0.506**	**-0.450**	**-0.464**	**0.529**
	p-value	0.085	0.248	0.097	0.019	0.041	0.034	0.014
Histological fibrosis scores in antimesenteric border region	***r***	**0.264**	**0.254**	**-0.292**	**0.557**	**-0.271**	**-0.278**	**0.490**
	p-value	0.248	0.267	0.199	0.009	0.235	0.223	0.024

Note: *MRE* Magnetic resonance enterography; *MTR* Magnetization transfer ratio; *ADC* apparent diffusion coefficient; *T2WI* T2-weighted imaging; *DWI* diffusion-weighted imaging.

Supplementary Table S5: 95% confidence interval of AUROCs for univariable MRE models in the three regions in surgical Cohort 1

Univariable MRE model	Subregion	95% CI (AUROC)
Normalized MTR	Mesenteric	0.73-0.95
	Antimesenteric	0.68-0.87
	Whole-circle	0.54-0.78
ADC	Mesenteric	0.69-0.94
	Antimesenteric	0.57-0.79
	Whole-circle	0.50-0.74
Pre-enhanced T1 value	Mesenteric	0.35-0.75
	Antimesenteric	0.38-0.62
	Whole-circle	0.51-0.74
Post-enhanced T1 value	Mesenteric	0.63-0.86
	Antimesenteric	0.61-0.84
	Whole-circle	0.51-0.76
T2WI signal	Mesenteric	0.48-0.86
	Antimesenteric	0.25-0.52
	Whole-circle	0.47-0.73
DWI signal	Mesenteric	0.47-0.92
	Antimesenteric	0.46-0.74
	Whole-circle	0.42-0.68

Note: *MRE* Magnetic resonance enterography; *CI* confidence interval; *AUROC* area under the receiver operating characteristic curve; *MTR* Magnetization transfer ratio; *ADC* apparent diffusion coefficient; *T2WI* T2-weighted imaging; *DWI* diffusion-weighted imaging.

Supplementary Table S6: Delong test for comparisons in AUROCs between univariable and multivariable MRE models in mesenteric border region in surgical Cohort 1

	T2WI signal	DWI signal	ADC	Normalized MTR	Pre-enhanced T1 value	Post-enhanced T1 value	Model 1	Model 2	Model 3	Model 4	Model 5
T2WI signal	1	0.3631	0.0697	0.0356	0.2183	0.996	0.7357	0.0918	0.0106	0.0028	0.0037
DWI signal	0.3631	1	0.4105	0.1632	0.0483	0.4548	0.4908	0.0135	0.004	0.0008	0.0095
ADC	0.0697	0.4105	1	0.553	0.015	0.1884	0.3881	0.6509	0.1225	0.0146	0.0221
Normalized MTR	0.0356	0.1632	0.553	1	0.0047	0.0898	0.2259	0.8	0.4055	0.1475	0.2662
Pre-enhanced T1 value	0.2183	0.0483	0.015	0.0047	1	0.0989	0.148	0.0085	0.0023	0.0007	0.0006
Post-enhanced T1 value	0.996	0.4548	0.1884	0.0898	0.0989	1	0.7458	0.1346	0.0418	0.01	0.0161
Model 1	0.7357	0.4908	0.3881	0.2259	0.148	0.7458	1	0.0902	0.0738	0.0401	0.0793
Model 2	0.0918	0.0135	0.6509	0.8	0.0085	0.1346	0.0902	1	0.2228	0.0362	0.1197
Model 3	0.0106	0.004	0.1225	0.4055	0.0023	0.0418	0.0738	0.2228	1	0.0495	0.3962
Model 4	0.0028	0.0008	0.0146	0.1475	0.0007	0.01	0.0401	0.0362	0.0495	1	0.6313
Model 5	0.0037	0.0095	0.0221	0.2662	0.0006	0.0161	0.0793	0.1197	0.3962	0.6313	1

Note: *ADC* apparent diffusion coefficient; *DWI* diffusion-weighted imaging; *T2WI* T2-weighted imaging; *MTR* Magnetization transfer ratio

Supplementary Table S7: Delong test for comparisons in AUROCs between univariable and multivariable MRE models in the antimesenteric region in surgical Cohort 1

	T2WI signal	DWI signal	ADC	Normalized MTR	Pre-enhanced T1 value	Post-enhanced T1 value	Model 1	Model 2	Model 3	Model 4	Model 5
T2WI signal	1	0.8378	0.2963	0.0016	0.1531	0.2766	0.3435	0.1409	0.0001	0.0001	<0.0001
DWI signal	0.8378	1	0.3557	0.0036	0.1178	0.2926	0.5287	0.1481	0.0002	0.0001	<0.0001
ADC	0.2963	0.3557	1	0.0665	0.0508	0.7792	0.4525	0.3791	0.035	0.0277	0.0105
Normalized MTR	0.0016	0.0036	0.0665	1	0.0006	0.217	0.0068	0.1139	0.7142	0.59	0.1929
Pre-enhanced T1 value	0.1531	0.1178	0.0508	0.0006	1	0.0223	0.0985	0.0338	0.0005	0.0003	<0.0001
Post-enhanced T1 value	0.2766	0.2926	0.7792	0.217	0.0223	1	0.3825	0.9206	0.1559	0.0755	0.0366
Model 1	0.3435	0.5287	0.4525	0.0068	0.0985	0.3825	1	0.2192	0.0004	0.0003	<0.0001
Model 2	0.1409	0.1481	0.3791	0.1139	0.0338	0.9206	0.2192	1	0.0565	0.0439	0.0176
Model 3	0.0001	0.0002	0.035	0.7142	0.0005	0.1559	0.0004	0.0565	1	0.6897	0.1958
Model 4	0.0001	0.0001	0.0277	0.59	0.0003	0.0755	0.0003	0.0439	0.6897	1	0.2045
Model 5	<0.0001	<0.0001	0.0105	0.1929	<0.0001	0.0366	<0.0001	0.0176	0.1958	0.2045	1

Note: *ADC* apparent diffusion coefficient; *DWI* diffusion-weighted imaging; *T2WI* T2-weighted imaging; *MTR* magnetization transfer ratio

Supplementary Table S8: Delong test for comparisons in AUROCs between univariable and multivariable MRE models in the whole-circle region in surgical Cohort 1

	T2WI signal	DWI signal	ADC	Normalized MTR	Pre-enhanced T1 value	Post-enhanced T1 value	Model 1	Model 2	Model 3	Model 4	Model 5
T2WI signal	1	0.034	0.4066	0.1352	0.6515	0.6171	0.0833	0.4417	0.01	0.0082	0.0012
DWI signal	0.034	1	0.6663	0.7707	0.6458	0.617	0.4989	0.6793	0.0699	0.0616	0.0078
ADC	0.4066	0.6663	1	0.5422	0.9042	0.9143	0.5904	0.9579	0.1837	0.1669	0.0614
Normalized MTR	0.1352	0.7707	0.5422	1	0.5294	0.5497	0.9243	0.6549	0.1213	0.1592	0.0618
Pre-enhanced T1 value	0.6515	0.6458	0.9042	0.5294	1	0.9789	0.5533	0.8525	0.1437	0.1341	0.0133
Post-enhanced T1 value	0.6171	0.617	0.9143	0.5497	0.9789	1	0.5323	0.8666	0.1528	0.0723	0.0236
Model 1	0.0833	0.4989	0.5904	0.9243	0.5533	0.5323	1	0.4774	0.1142	0.107	0.0127
Model 2	0.4417	0.6793	0.9579	0.6549	0.8525	0.8666	0.4774	1	0.0655	0.0723	0.0161
Model 3	0.01	0.0699	0.1837	0.1213	0.1437	0.1528	0.1142	0.0655	1	0.9348	0.2686
Model 4	0.0082	0.0616	0.1669	0.1592	0.1341	0.0723	0.107	0.0723	0.9348	1	0.2101
Model 5	0.0012	0.0078	0.0614	0.0618	0.0133	0.0236	0.0127	0.0161	0.2686	0.2101	1

Note: *ADC* apparent diffusion coefficient; *DWI* diffusion-weighted imaging; *T2WI* T2-weighted imaging; *MTR* magnetization transfer ratio

Supplementary Table S9: Delong test between each multivariable model in the mesenteric border, antimesenteric border, and whole-circle region (none-mild fibrosis vs. moderate-severe fibrosis lesions) in surgical Cohort 1

		Mesenteric	Antimesenteric	Whole-circle
Model 1	Mesenteric	1	0.2321	0.2953
	Antimesenteric	0.2321	1	0.7833
	Whole-circle	0.2953	0.7833	1
Model 2	Mesenteric	1	0.0639	0.0102
	Antimesenteric	0.0639	1	0.4515
	Whole-circle	0.0102	0.4515	1
Model 3	Mesenteric	1	0.2292	0.0337
	Antimesenteric	0.2292	1	0.2954
	Whole-circle	0.0337	0.2954	1
Model 4	Mesenteric	1	0.1269	0.0115
	Antimesenteric	0.1269	1	0.2565
	Whole-circle	0.0115	0.2565	1
Model 5	Mesenteric	1	0.3031	0.0643
	Antimesenteric	0.3031	1	0.3248
	Whole-circle	0.0643	0.3248	1

Supplementary Figures

Supplementary Fig. S1 The quantitative evaluation of collagen in the mesenteric border, antimesenteric border, and whole-circle bowel walls.

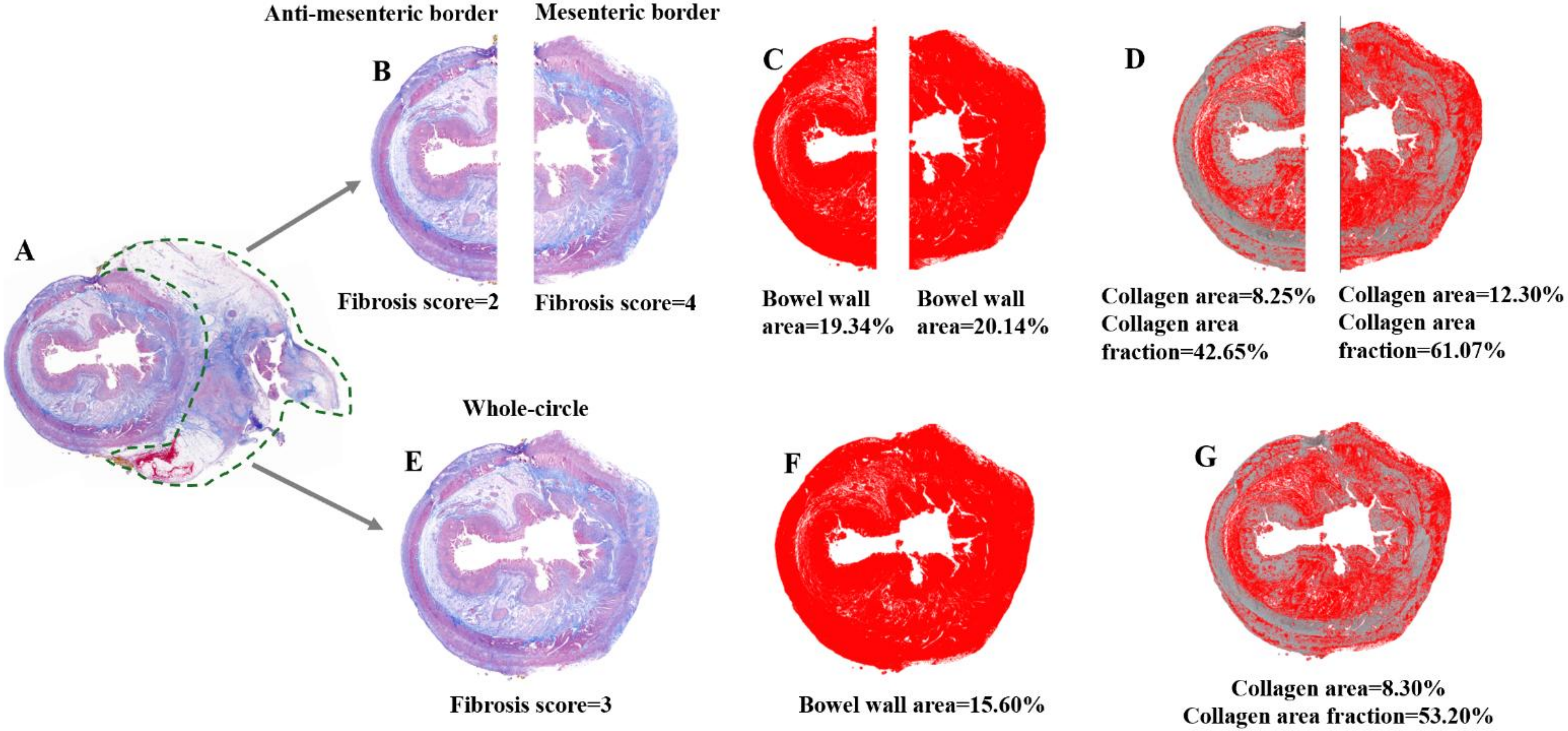

Supplementary Fig. S1. The quantitative evaluation of collagen in the mesenteric border, antimesenteric border, and whole-circle bowel walls. (A) The Masson trichrome-stained images of the whole-circle and whole-slide intestinal wall (×20 magnification), obtained from a 32-year-old male patient with Crohn disease, are converted into PNG format at 0.4x, using CaseViewer (version 2.4; 3DHISTECH Ltd.; H-1141 Budapest, Ov u.3, Hungary). The division of the mesenteric border and antimesenteric border is determined by the extent of creeping fat wrapping around the gut (indicated by green dotted line). According to this demarcation, the Masson trichrome stained images are manually segmented into two separate images, representing the mesenteric border wall and antimesenteric border wall (B-D). Simultaneously, any presence of creeping fat on the images of the mesenteric border wall is also eliminated using Adobe Photoshop software (version: 21.2.3) (B-D). Subsequently, the radiologist (J.J.L, responsible for conducting MRI *ex vivo* scans and 3D printing molds but unaware of fibrosis score results) employs *ImageJ* software (v1.50i; National Institutes of Health, USA) to calculate the collagen area fraction in three regions using a reported approach [7], thereby quantifying the amount of collagen. For example, in calculating the collagen level in mesenteric border region on Masson trichrome-stained image (B), the area of mesenteric border region (indicating as red area) is divided by the visual field area (including the white background and red bowel wall) to automatically yield the area ratio of mesenteric border region (C). Subsequently, collagen (indicating as red area) in the mesenteric border region is automatically identified and differentiated from other tissue properties (indicating as grey area) after converting the color images into grey-colored figures (D). Similarly, the area of collagen is divided by the area of the visual field area to automatically yield the area ratio of collagen. Hence, the ratio between the area of the collagen and the total area of the bowel wall in mesenteric border region is calculated as follows: Collagen area fraction [%] = [Area ratio of collagen/Area ratio of the bowel wall] × 100%. Using the same methodology, the collagen area fractions of the antimesenteric border region (B-D) and the whole-circle region (E-G) are calculated.

Supplementary Fig. S2 The measurement of α-SMA⁺ area fractions and staining intensity at submucosa in the mesenteric border, antimesenteric border, and whole-circle bowel walls.

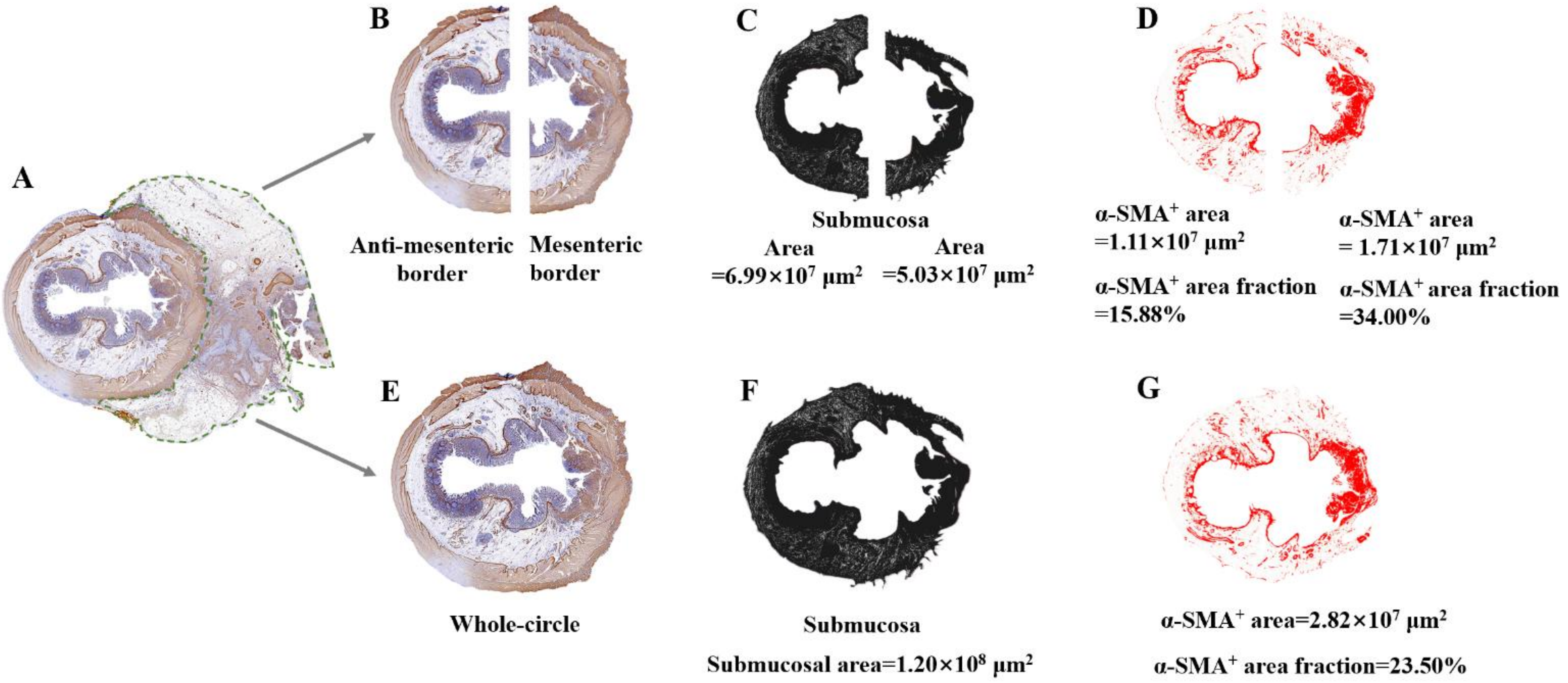

Supplementary Fig. S2. The measurement of α-SMA$^+$ area fractions and staining intensity at submucosa in the mesenteric border, antimesenteric border, and whole-circle bowel walls. (A) The α-SMA-stained immunohistochemistry images of the whole-circle and whole-slide intestinal wall (×20 magnification) are acquired from the same resected bowel segment, originating from the same patient as depicted in Supplementary Fig. S1. The division of the mesenteric and antimesenteric borders (B-D) remains consistent with Supplementary Fig. S1. The radiologist (W.K.Z)

manually segments the submucosa (indicating as the black area) and calculates its area in each subregion (C). In the submucosa, the α-SMA$^+$ area (indicating as the red area) and the α-SMA$^+$ integrated density (IntDen$^{α\text{-}SMA}$) are also automatically extracted using *ImageJ* software (D). Ultimately, the submucosal α-SMA$^+$ area fraction in mesenteric or antimesenteric border region is calculated as follows: α-SMA$^+$ area fraction [%] = [Submucosal α-SMA$^+$ area / Submucosal area] × 100 %. At the same time, the mean α-SMA$^+$ staining intensity in mesenteric or antimesenteric border region is calculated as follows: Mean α-SMA$^+$ staining intensity = IntDen$^{α\text{-}SMA}$ / Submucosal area. Similarly, the α-SMA$^+$ area fraction and the mean α-SMA$^+$ staining intensity in the whole-circle region (E-G) are calculated.

Supplementary Fig. S3 The measurement of thickness of the muscularis propria in the mesenteric border, antimesenteric border, and whole-circle bowel walls.

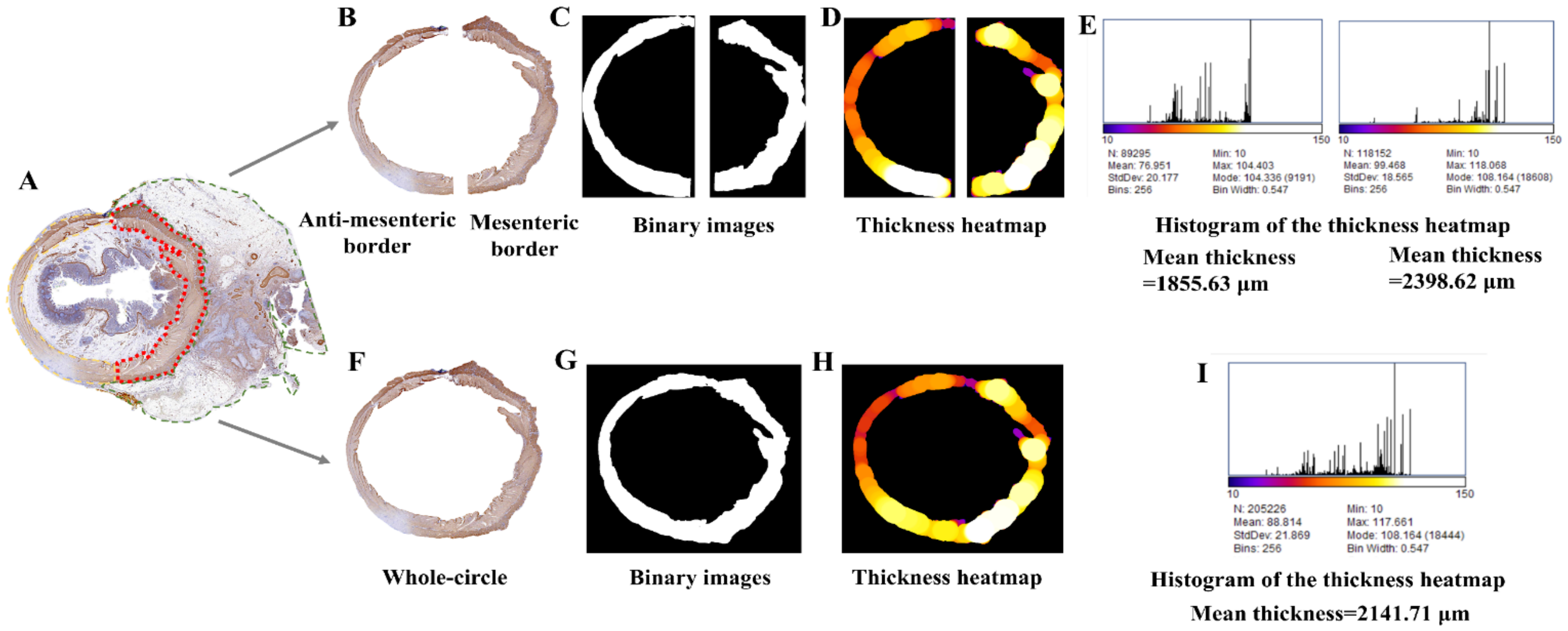

Supplementary Fig. S3. The measurement of thickness of the muscularis propria in the mesenteric border, antimesenteric border, and whole-circle bowel walls. (A) The α-SMA-stained immunohistochemistry images of the whole-circle and whole-slide intestinal wall (×20 magnification) are acquired from the same resected bowel segment, originating from the same patient as depicted in **Supplementary Fig. S1 and S2**. The division of the mesenteric border and antimesenteric border (B-D) remains consistent with the aforementioned two cases. (B)The muscularis propria is manually segmented using *ImageJ* software by the radiologist (W.K.Z). Subsequently, the image of muscularis propria is binarized (C; indicating as the white area) and processed using the automatic algorithm in *ImageJ* to generate a thickness heatmap (D). Specifically, local thickness function in *ImageJ* plugin [8]

was used to achieve this calculation. The basic logic of the local thickness function [9] is as follows: the muscularis propria region, defined as the white area in the binarized image, is denoted as Ω ($\Omega \in R^2$). The local thickness is defined as the diameter of the largest circle centered at a point *p*, where the circle is entirely contained within Ω. The radius of the maximum circle is the shortest distance from point *p* to the boundary of the muscularis propria region, and all parts of this circle must lie within this region. A set of points *P* is sampled within Ω to generate a collection of circles. These circles are used to construct a thickness heatmap (D) where the color gradient ranges from purple to white, indicating an increase in the diameter of the circles. By performing statistical analysis on these local thicknesses, a histogram (E) is produced, from which the average, extreme values, and other statistical data of the muscularis propria thickness can be derived. Using the same methodology, the mean thickness of the muscularis propria in the whole-circle region is calculated (F-I).

Supplementary Fig. S4 SHAP plots illustrate the contribution of each MRE parameter to the model's prediction in the (**a**) mesenteric border, (b) antimesenteric border, and (**c**) whole-circle regions.

These plots reveal that normalized MTR was the most influential driver for predicting moderate-to-severe fibrosis in the mesenteric border model, whereas its importance was reduced in other regions.

Supplementary Fig. S4a: The optimal model (model 4) for mesenteric border region

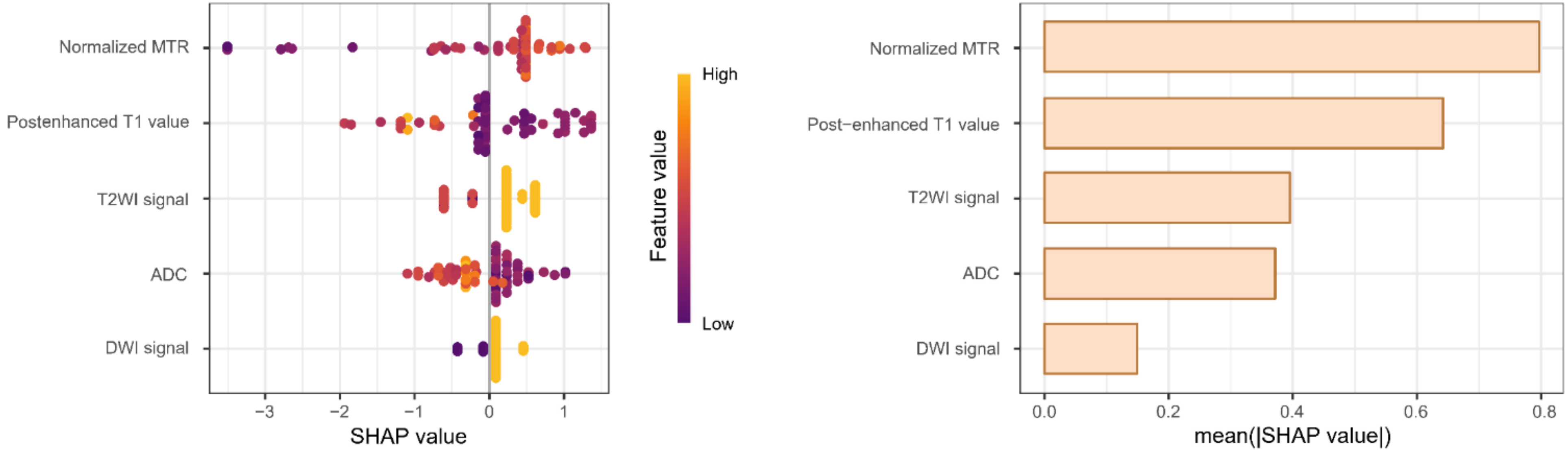

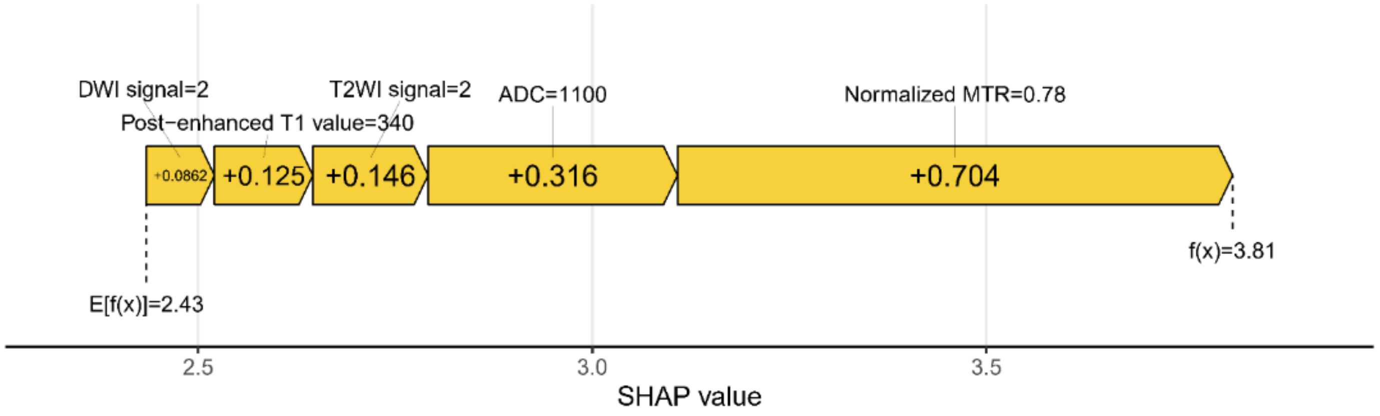

Supplementary Fig. S4b: The optimal model (model 5) for antimesenteric border region

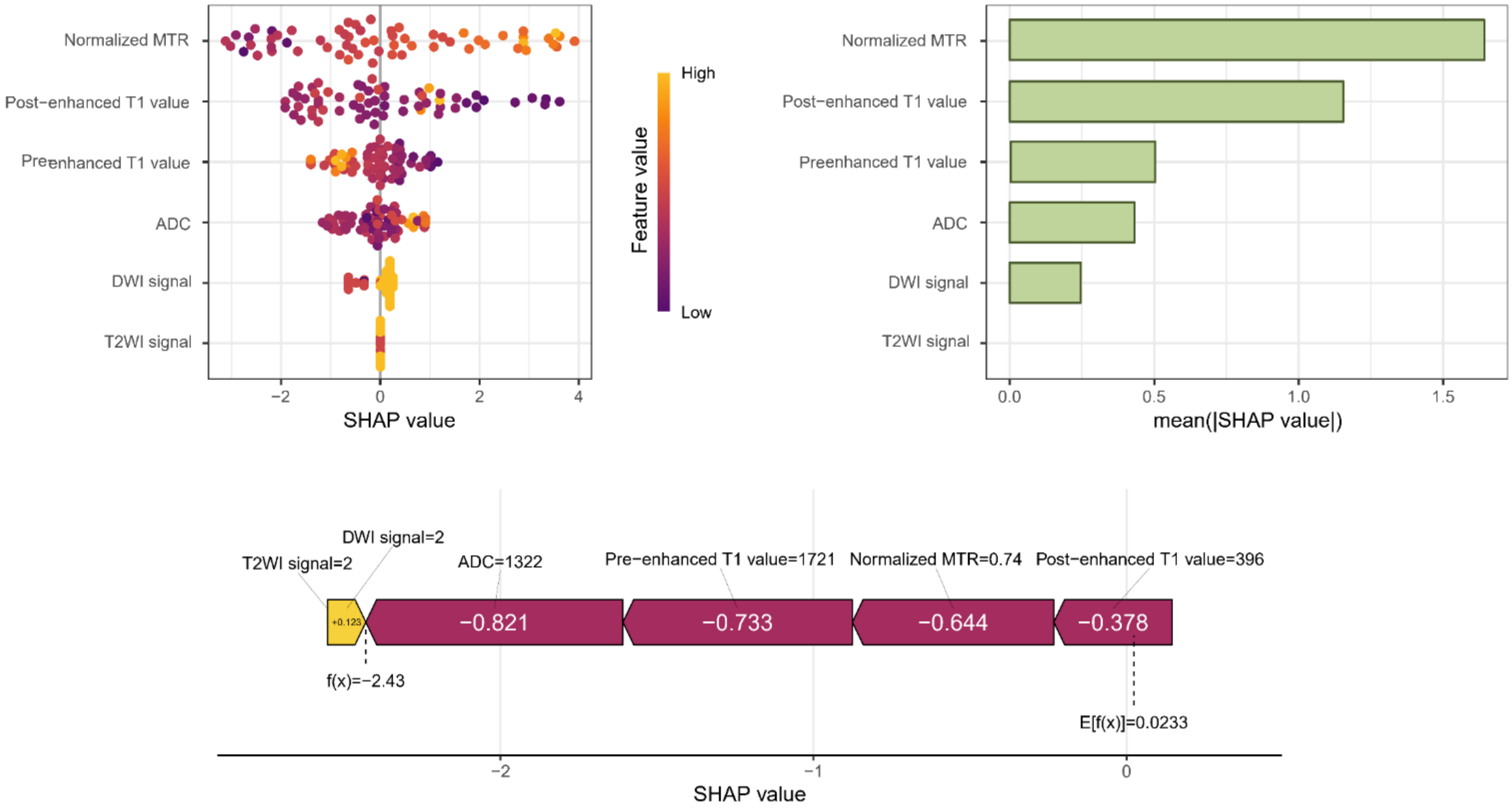

Supplementary Fig. S4c: The optimal model (model 5) for whole-circle region

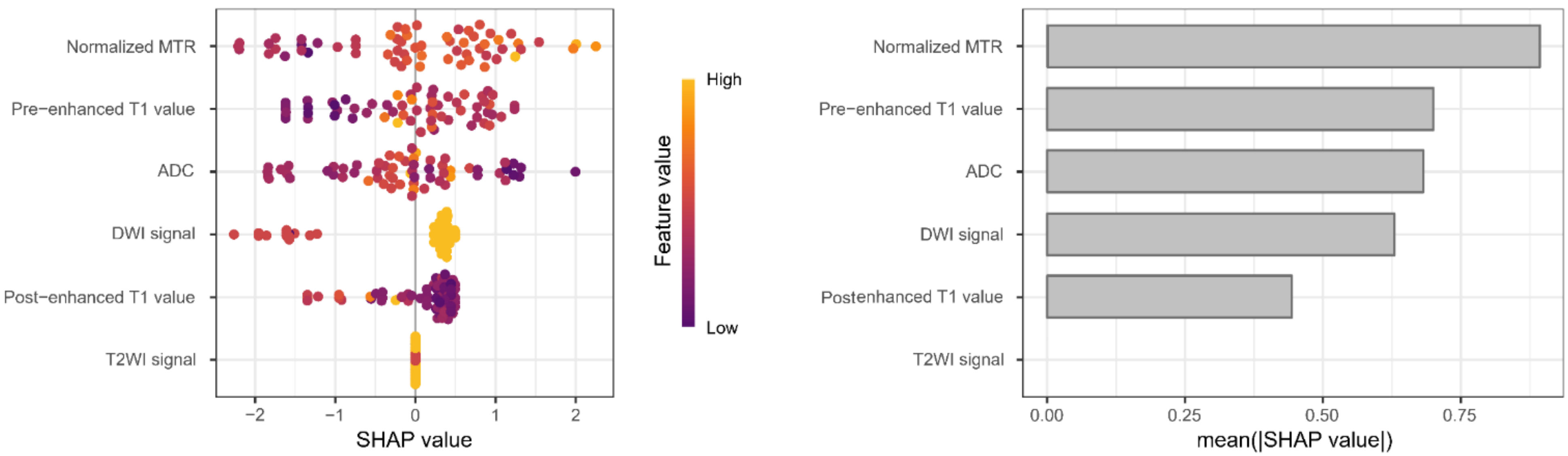

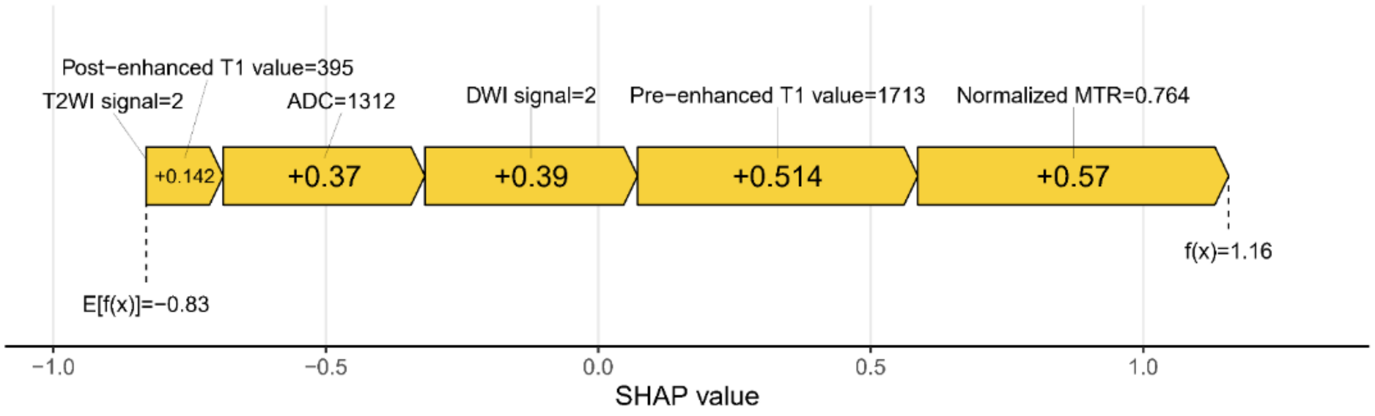

Supplementary Fig. S4. SHapley Additive exPlanations (SHAP) illustrating the imaging features used to develop the optimal model for the mesenteric border (a), antimesenteric border (b), and whole-circle (c) regions. The importance of the imaging features is demonstrated in a descending order (top left plot), with variable weights illustrating their importance (top right plot). In the top left image, the horizontal location indicates whether the effect of that value is associated with a higher or lower prediction, and the color represents the level of that variable (high=yellow; low=purple) for that observation. For example, in the optimal model for mesenteric border region (A), the increase in normalized MTR or the decrease in post-enhanced T1 value has a positive impact on the model's prediction result, driving it towards predictive result associated with moderate-to-severe intestinal fibrosis. SHAP force plots (below) derived from the representative CD patients described in **Figure.6** of the main text illustrate how these models predict the probability of intestinal fibrosis in the corresponding regions. In the force plots, variables that increase the model's predictive probability towards moderate-severe fibrosis are shown on the left in yellow, while those that decrease it are shown on the right in purple. The actual values of the variables are shown alongside the variables' name. The (f(x)) is the probabilistic predicted value. The length of the arrows visually represents the effect on the prediction. Longer arrow indicates larger effect.

Supplementary Fig. S5 Workflow and input/output structure of the APP-based calculator for intestinal fibrosis.

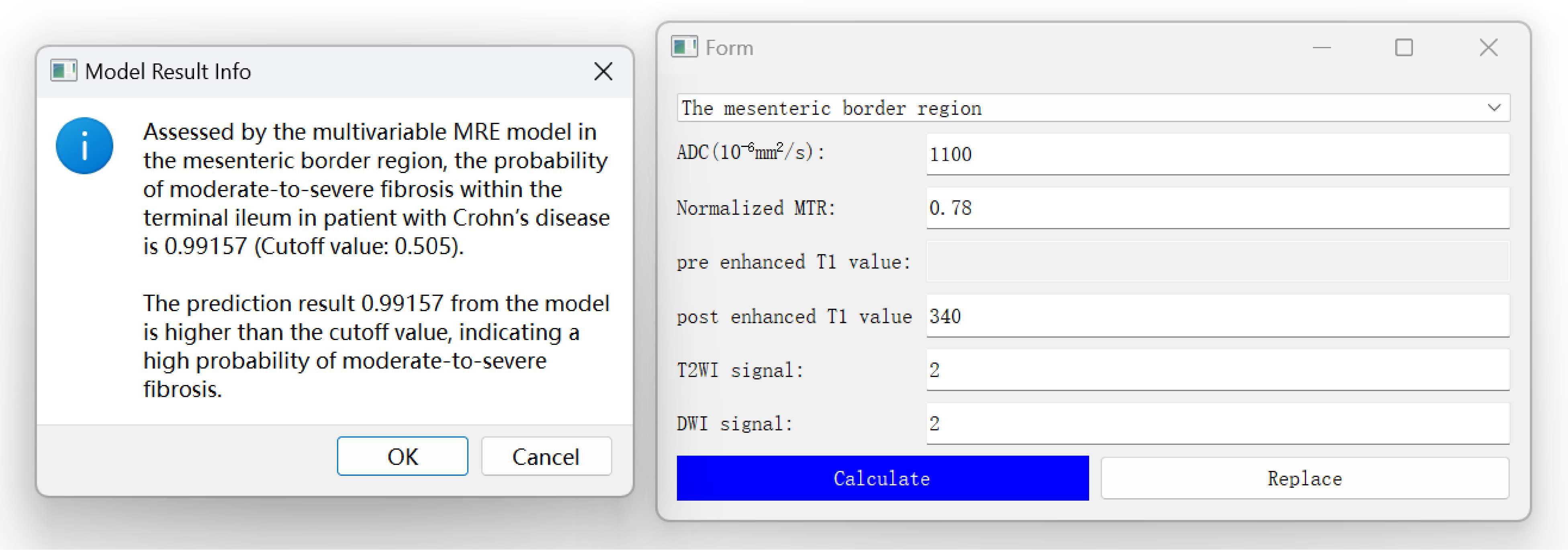

Supplementary Fig. S5. Workflow and input/output structure of the APP-based calculator for intestinal fibrosis. The open-access application (APP) interface. **Inputs:** Select the target intestinal region (Mesenteric, Antimesenteric, or Whole-circle). Users are required to input the six MRE parameters (T2WI signal, DWI signal, ADC, normalized MTR, pre-enhanced T1 value, and post-enhanced T1 value) measured in the region of interest (e.g., mesenteric border region, as shown here). **Outputs:** The APP automatically computes and displays the prediction of moderate-to-severe fibrosis and provides a categorical classification (e.g., "none-to-mild fibrosis" or "moderate-to-severe fibrosis") based on the pre-validated threshold. Also, a representative case (same patient as in **Figure 6**) demonstrating the input of parameters and the resultant output for the mesenteric border region. The predictions for antimesenteric border region and whole-circle region can also be performed using this APP-based calculator. The calculator is available for download at https://github.com/SchwarzW/APP-based-calculator-for-assessing-fibrosis-in-different-intestinal-subregions.

Appendix: Overview of All Scoring Systems and Parameters

This table serves as a quick reference for all scoring systems and parameters utilized in this article.

An overview of the scoring systems or parameters utilized in this article

**Scoring system**	**Interpretation**	**References**
**Imaging**		
Mesenteric creeping fat index (MCFI)	An imaging index scored from 1 to 8 to grade creeping fat wrapping around the gut by assessing the extent of bowel circumference encompassed by vessels in fat *in vivo*. Specifically, intestinal circumference was divided into eight equal zones, and a score of 1 was assigned to each zone overlapped by mesenteric vessels.	J Crohns Colitis. 2021;15(7):1161-73
Reconstruction quality score of MCFI	Score 0: low quality, with blurred mesenteric vessels and an indistinguishable mesenteric border region.<br>Score 1: moderate quality, where the mesenteric vessels were visible and the mesenteric border region could be identified.<br>Score 2: high quality, with clearly visible mesenteric vessels and accurate identification of the mesenteric border region.	/
T2-weighted imaging (T2WI) signal	Score 0: isointensity compared to adjacent normal bowel wall.<br>Score 1: slightly increased intensity with dark grey appearance.<br>Score 2: significantly increased intensity with white gray or white appearance.	Eur Radiol. 2012;22(11):2494-501.
Diffusion-weighted imaging (DWI) signal	Score 0: isointensity compared to adjacent normal bowel wall.<br>Score 1: slightly increased intensity compared to adjacent normal bowel wall.<br>Score 2: significantly increased intensity compared to adjacent normal bowel wall.	Inflamm Bowel Dis. 2017;23(2):244-53

Normalized magnetization transfer ratio (MTR)	The MTR of bowel wall is divided by the MTR of muscle, using the following formula: $\text{Normalized MTR} = \frac{\text{MTR bowel wall}}{\text{MTR psoas muscle}}$.	Radiology. 2018;287(2):494-503.
The apparent diffusion coefficient (ADC)	Direct measurement of ADC values of bowel walls using RadiAnt DICOM Viewer	/
Pre-/post-enhanced T1 value	Direct measurement of T1 values of bowel walls using RadiAnt DICOM Viewer	Biosensors (Basel). 2021;11(9).
**Specimen**		
Fat wrapping score-specimen	It is scored from 1 to 8 based on the overlap of creeping fat with each zone in intestinal circumference divided into eight equal parts, similar to the MCFI scoring system.	J Crohns Colitis. 2021;15(7):1161-73
**Pathology**		
Histologic fibrosis scores	Score 0: no fibrosis.<br>Score 1: minimal fibrosis in submucosa or subserosa.<br>Score 2: increased submucosal fibrosis, septa into muscularis propria.<br>Score 3: septa through muscularis propria, increase in subserosal collage.<br>Score 4: significant transmural scar, marked subserosal collagen.<br>Scores 0-2: none-mild disease.<br>Scores 3-4: moderate-severe disease.	Inflammatory Bowel Diseases. 2012;18(5):849-56.<br><br>Gastroenterology. 2021;160(7):2303-16.e11.<br><br>Eur Radiol. 2019;29(6):3069-78.
Collagen area fraction	Collagen area fraction [%] = [Area ratio of collagen/Area ratio of the bowel wall] × 100%	BMC Gastroenterol. 2019;19(1):180.
α-SMA$^+$ area fraction	α-SMA$^+$ area fraction [%] = [Submucosal α-SMA$^+$ area / Submucosal area] × 100 %	/

α-SMA⁺ staining intensity	α-SMA⁺ staining intensity = $IntDen^{α\text{-}SMA}$ / Submucosal area.<br>$IntDen^{α\text{-}SMA}$: integrated density	/
Mean thickness of the muscularis propria	The mean thickness of the muscular propria is calculated using the automatic algorithm in ImageJ (detailed in Supplementary Figure.3)	Biochim Biophys Acta Mol Cell Res. 2022;1869(9):119286.<br><br>Journal of Microscopy. 1997;185(1):67-75

Reference

1 Li X-H, Feng S-T, Cao Q-H et al (2021) Degree of Creeping Fat Assessed by Computed tomography enterography is associated with intestinal fibrotic stricture in patients with Crohn’s disease: a potentially novel mesenteric creeping fat index. J Crohns Colitis 15:1161–1173. https://doi.org/10.1093/ecco-jcc/jjab005

2 Menys A, Atkinson D, Odille F et al (2012) Quantified terminal ileal motility during MR enterography as a potential biomarker of Crohn's disease activity: a preliminary study. Eur Radiol 22:2494–2501. https://doi.org/10.1007/s00330-012-2514-2

3 Li XH, Sun CH, Mao R et al (2017) Diffusion-weighted MRI Enables to accurately grade inflammatory activity in patients of ileocolonic Crohn's disease: results from an observational study. Inflamm Bowel Dis 23:244–253. https://doi.org/10.1097/MIB.0000000000001001

4 Li XH, Mao R, Huang SY et al (2018) Characterization of degree of intestinal fibrosis in patients with Crohn disease by using magnetization transfer MR imaging. Radiology 287:494–503. https://doi.org/10.1148/radiol.2017171221

5 Dwivedi DK, Chatzinoff Y, Zhang Y et al (2018) Development of a patient-specific tumor mold using magnetic resonance imaging and 3-dimensional printing technology for targeted tissue procurement and radiomics analysis of renal masses. Urology 112:209-214. https://doi.org/10.1016/j.urology.2017.08.056

6 Turkbey B, Mani H, Shah V et al (2011) Multiparametric 3T prostate magnetic resonance imaging to detect cancer: histopathological correlation using prostatectomy specimens processed in customized magnetic resonance imaging based molds. J Urol 186:1818–1824. https://doi.org/10.1016/j.juro.2011.07.013

7 Li XH, Fang ZN, Guan TM et al (2019) A novel collagen area fraction index to quantitatively assess bowel fibrosis in patients with Crohn's disease. BMC Gastroenterol 19:180. https://doi.org/10.1186/s12876-019-1100-3

8 Urbaniak P, Wronski S, Tarasiuk J, Lipinski P, Kotwicka M (2022) A new method to estimate 3D cell parameters from 2D microscopy images. Biochim Biophys Acta Mol Cell Res 1869:119286. https://doi.org/10.1016/j.bbamcr.2022.119286

9 Hildebrand T, Rüegsegger P (1997) A new method for the model-independent assessment of thickness in three-dimensional images. Journal of Microscopy 185:67–75. https://doi.org/10.1046/j.1365-2818.1997.1340694.x
